# Coupling optogenetic stimulation with NanoLuc-based luminescence (BRET) Ca^++^ sensing

**DOI:** 10.1038/ncomms13268

**Published:** 2016-10-27

**Authors:** Jie Yang, Derrick Cumberbatch, Samuel Centanni, Shu-qun Shi, Danny Winder, Donna Webb, Carl Hirschie Johnson

**Affiliations:** 1 Department of Biological Sciences, Vanderbilt University, Nashville, Tennessee 37235-1634, USA; 2 Department of Molecular Physiology and Biophysics, Vanderbilt University, Nashville, Tennessee 37235-1634, USA

## Abstract

Optogenetic techniques allow intracellular manipulation of Ca^++^ by illumination of light-absorbing probe molecules such as channelrhodopsins and melanopsins. The consequences of optogenetic stimulation would optimally be recorded by non-invasive optical methods. However, most current optical methods for monitoring Ca^++^ levels are based on fluorescence excitation that can cause unwanted stimulation of the optogenetic probe and other undesirable effects such as tissue autofluorescence. Luminescence is an alternate optical technology that avoids the problems associated with fluorescence. Using a new bright luciferase, we here develop a genetically encoded Ca^++^ sensor that is ratiometric by virtue of bioluminescence resonance energy transfer (BRET). This sensor has a large dynamic range and partners optimally with optogenetic probes. Ca^++^ fluxes that are elicited by brief pulses of light to cultured cells expressing melanopsin and to neurons-expressing channelrhodopsin are quantified and imaged with the BRET Ca^++^ sensor in darkness, thereby avoiding undesirable consequences of fluorescence irradiation.

## Abstract (web summary)

The coupling of optogenetics with fluorescent Ca^2+^ sensors is confounded by sensitivity of optogenetic probes to light used to excite the sensors. Here the authors develop a Ca^2+^ sensor based on bioluminescence resonance energy transfer (BRET) that monitors Ca^2+^ fluxes in darkness without excitation.

New optogenetic methods for stimulating calcium ion (Ca^++^) fluxes and neural activity are revolutionizing cellular and neurobiological research^1^,^2^,^3^. Brief exposure to blue–green light of cells expressing channelrhodopsin or melanopsin can elicit excitatory cation fluxes (channelrhodopsin-2, that is, ChR2) or Ca^++^ fluxes (melanopsin)^2^,^3^,^4^,^5^. In many applications, these optogenetic methods would optimally be partnered with a minimally invasive optical method to directly monitor the impact of the perturbation. For example, optical measurements of neural activity before and after optogenetic stimulation could facilitate recording in freely behaving animals *in vivo* and/or in multiple neurons simultaneously. While there are optical methods for measuring neural activity that use fluorescence sensors for synaptic vesicle pH (refs 6, 7, 8, 9, 10, 11, 12), genetically encoded Ca^++^ sensors targeted to the presynaptic terminal and/or dendritic spines that signal the large Ca^++^ transient in response to an action potential are currently the most promising technique. In particular, the GCaMP family of reporters has undergone considerable optimization by structural design and mutagenesis/screening^7^,^8^,^9^.

However, fluorescent sensors for measuring [Ca^++^] and/or neural activity are unfavorable for use with optogenetic probes because the continuous excitation that is required to monitor the sensor can (i) trigger tissue autofluorescence and phototoxicity/photoresponses, (ii) only poorly penetrate tissue and (iii) photobleach the probe/sensor. Moreover, optogenetic probes are often directly stimulated by the irradiation used to excite the fluorescent sensor of pH, Ca^++^ or membrane voltage. Some of the new generation sensors can be excited by longer wavelength light (for example, R-GECO1 (ref. 13)) to lessen cross-excitation, but the ‘tails’ of stimulation by light absorbing pigments render the total exclusion of cross-talk impossible. While two-photon technology in conjunction with fluorescence sensors is under very active development to circumvent some of these problems (for example, autofluorescence and probe crosstalk)^7^,^8^, this technology is expensive and can photobleach probes with the high-order photon flux (as well as damaging the cells/tissues themselves)^14^. A totally novel strategy for an optical sensor that is independent of light excitation is worthy of consideration as a partner with optogenetic stimulation.

A completely different approach for an optical sensor of [Ca^++^] and/or neural activity would be a luminescence sensor that does not require excitation^15^ because the light signal is a result of an enzyme-catalysed chemical reaction rather than fluorescence. Therefore, the optical measurement of activity is made in darkness, and brief pulses of light would be dedicated specifically to stimulate the optogenetic probe. Because there is no continuous excitation and biological activities are assessed in darkness, there is no autofluorescence, photobleaching, phototoxicity or photoresponse. An optimal luminescence sensor would be ion-specific, sensitive, genetically encodable and ratiometric. The desirability of the first three properties is obvious, but regarding the final optimal property, ratiometric sensors monitor an ionic concentration independently of the intracellular concentration of the sensor (that is, expression level for a genetically encoded sensor) because the measured quantity is the ratio of two wavelengths. Ratiometric recording is less influenced by changes in optical path length, and it also helps to cancel movement and growth artifacts^16^. The currently available genetically encodable luminescent probes for Ca^++^ (for example, aequorin, obelin, Nano-lantern(Ca^2+^)) have desirable properties in terms of specificity, sensitivity and encodability, but they are not ratiometric^17^,^18^,^19^. Moreover, while aequorin has been successfully used as a luminescent reporter of neural activity in zebrafish^20^, it has been less successful in mammalian neurons^17^,^18^. The studies with aequorin encourage the feasibility of using luminescent reporters of synaptic activity while underscoring that an optimal luminescent probe has not yet been developed for mammalian applications.

Based on ratiometric bioluminescence resonance energy transfer (BRET) technology^21^,^22^,^23^, we have developed a new Ca^++^ sensor to use in conjunction with optogenetic probes. BRET avoids the problems of fluorescence excitation (for example, stimulation of an optogenetic probe, autofluorescence, phototoxicity, poor tissue penetration and photobleaching) by monitoring in darkness the luminescence generated by enzymatic catalysis mediated by a luciferase. A newly developed luciferase (NanoLuc) is 100–150 × brighter than previous luciferases^24^, which greatly expands the usefulness of luminescence technology. Our Ca^++^ sensor is genetically encodable to allow targeting to specific cell types and/or cellular loci, and employs this bright new luciferase to obtain excellent signal strength. We show here that this BRET sensor ratiometrically reports Ca^++^ fluxes elicited by the optogenetic probes ChR2 and melanopsin in fibroblasts and hippocampal neurons.

## Results

### *In vitro* characterization of BRET Ca^++^ sensor

**Figure 1:**
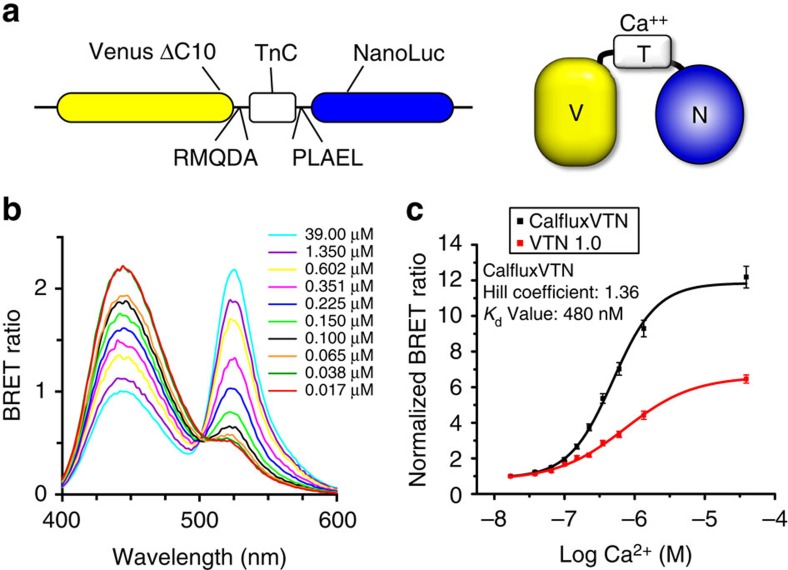
Characteristics of CalfluxVTN *in vitro.* (**a**) The troponin C domain (TnC) was inserted between Venus (with 10 amino acids deleted from the C terminus=CΔ10) and NanoLuc luciferase, using the indicated peptide linkers. (**b**) QuantaMaster spectrophotometer assays of luminescence/BRET spectra from purified CalfluxVTN protein *in vitro.* CalfluxVTN was purified via its 6-His tag and its emission spectrum from 400–600 nm was recorded in buffers with varying concentrations of free Ca^++^ (range is from 0.017 to 39 μM). Luminescence was initiated by the addition of furimazine substrate. (**c**) Comparison of [Ca^++^] dependency *in vitro* for the VTN 1.0 (red trace, see Supplementary Fig. 1p) versus CalfluxVTN (black trace, same as Supplementary Fig. 1a) versions of the Troponin-C moiety. For these QuantaMaster measurements, BRET ratio values (Venus/NanoLuc) were calculated by dividing the light emitted at the Venus peak (∼525 nm) by that emitted at the NanoLuc peak (∼450 nm), after a full scan of the light emission spectrum from 400 to 600 nm. Values were normalized by dividing each BRET ratio by the ratio at 0.017 μM Ca^++^. Values for the hill coefficient and *K*_d_ of Calflux VTN are shown in **c**. Mean±s.d., *n*=3.

Our sensor of free calcium ions shares the Ca^++^-sensitive troponin-C sequence (from *Opsanus tau*) and linkers with the fluorescent Ca^++^ indicators Twitch-2 and Twitch-3 (ref. 16). Our BRET sensor interposes this Ca^++^-sensitive troponin-C sequence between NanoLuc luciferase^24^ and a C-terminal truncated version of the fluorescent protein Venus^25^,^26^ with linkers shown in Fig. 1a. We call this Ca^++^ sensor CalfluxVTN for ‘CALcium FLUX composed of Venus (V), Troponin (T) and NanoLuc (N).’ The Ca^++^-sensitive troponin sequence undergoes a conformational change in response to binding Ca^++^ that brings NanoLuc closer to Venus so that resonance energy transfer can occur with a concomitant spectral shift^16^,^27^. The spectral shifts of the NanoLuc-catalysed luminescence as a function of [Ca^++^] *in vitro* are shown in Fig. 1b, where the 450 nm emission of NanoLuc is increasingly resonance-transferred to the 525 nm emission of Venus as the [Ca^++^] changes from 0.017 μM to 39 μM. Figure 1c plots the BRET ratio (525 nm Venus peak : 450 nm NanoLuc peak) as a function of [Ca^++^] *in vitro* for two different versions of CalfluxVTN. Because the fluorescent Twitch-3 version of CalfluxVTN exhibited a larger dynamic range within the range of Ca^++^ concentrations that are likely to be encountered in cells (approximately log −7 to −6 M Ca^++^, that is, 0.1–1 μM, Fig. 1c), our further experiments used this version to create the luminescent CalfluxVTN. Before this selection, however, we tested 15 combinations of native and circularly permuted Venus and NanoLuc (Supplementary Fig. 1); the selected version of CalfluxVTN (Fig. 1a) had the most desirable blend of brightness and dynamic BRET ratio change within the 0.1–1 μM [Ca^++^] range (Supplementary Fig. 1 and Supplementary Table 1).

**Figure 2:**
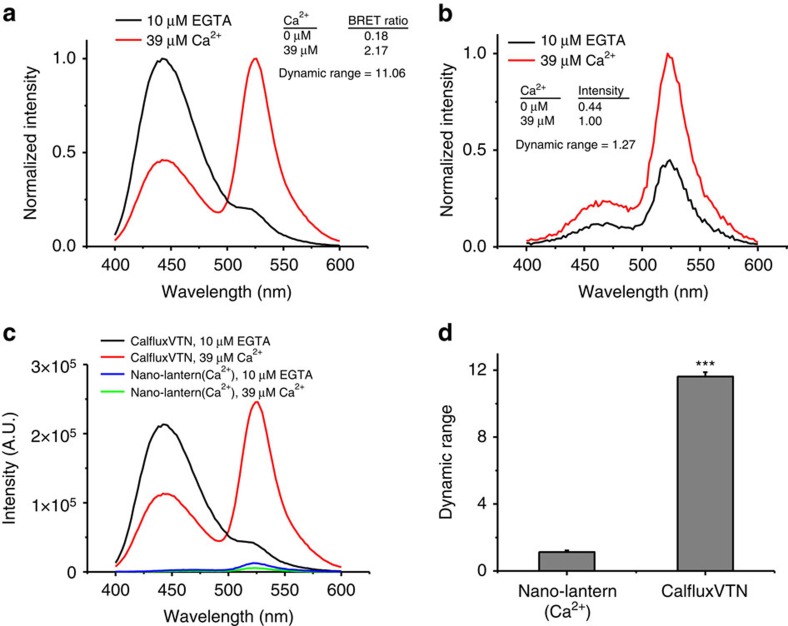
Comparison of the intensity and ratio changes of CalfluxVTN and Nano-lantern(Ca^2+^). (**a**) The normalized emission spectra of equal amounts of purified CalfluxVTN and (**b**) Nano-lantern(Ca^2+^) proteins. Each protein’s effective concentration was estimated by the intensity of its fluorescence signal under the same conditions. Spectra were recorded in 10 μM EGTA or 39 μM Ca^++^ solutions using a QuantaMaster spectrometer. (**c**) Plots of unnormalized spectra of CalfluxVTN and Nano-lantern(Ca^2+^) to compare relative intensities of each probe. (**d**) The values obtained in 10 μM EGTA or 39 μM Ca^++^ solutions were recorded and the maximum dynamic ranges were calculated. Dynamic range was calculated as [*R*−*R*_0_]/*R*_0_ (for CalfluxVTN) or as [*I*−*I*_0_]/*I*_0_ (for Nano-lantern(Ca^2+^)), where *R*, ratio and *I*, intensity. (Mean±s.d., *n*=3., ***P<0.001, unpaired *t*-test). a.u., arbitrary unit.

Before the development of CalfluxVTN, the brightest available luminescent sensor of Ca^++^ was Nano-lantern(Ca^2+^) (ref. 19). By virtue of its much brighter NanoLuc luciferase, CalfluxVTN is at least 30–50 × brighter than Nano-lantern(Ca^2+^)(Fig. 2a–c). Moreover, the dynamic range of CalfluxVTN *in vitro* from very low [Ca^++^] (∼0 μM) to high [Ca^++^] (∼39 μM) is more than an 11-fold change of BRET ratio (Fig. 2d), whereas the dynamic range of nano-lantern(Ca^2+^) is ∼1.3-fold in terms of its change of intensity in response to free calcium (Fig. 2d; the response of Nano-lantern(Ca^2+^) to [Ca^++^] is based on changes of luminescence intensity, whereas the response of CalfluxVTN is based on ratiometric changes in BRET efficiency). Moreover, Calflux not only has a strong signal (bright luminescence, large dynamic range), its response is also specific for Ca^++^; in contrast to its exquisite responsiveness to Ca^++^, the BRET ratio of CalfluxVTN is not sensitive to changes of Mg^++^, pH, K^+^ or Na^+^ that might be expected to occur within physiological ranges within cells (Supplementary Fig. 2).

### BRET Ca^++^ sensor expressed in cell cultures

**Figure 3:**
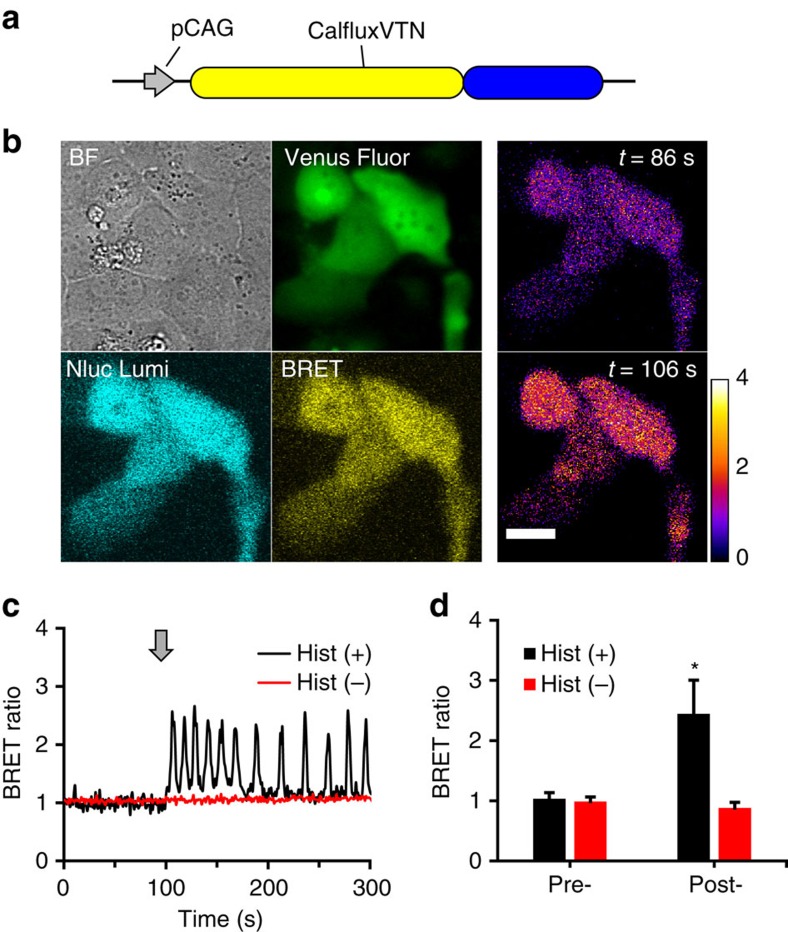
CalfluxVTN as an *in vivo* Ca^++^ sensor in mammalian cells. (**a**) CalfluxVTN construct driven by CAG promoter (pCAG^37^) for mammalian cell expression. (**b**) Photomicrographs of HeLa cells expressing CalfluxVTN. The four leftmost panels are as follows: BF, bright-field image; Venus Fluor, Venus fluorescence image in pseudo-colour green; Nluc Lumi, luminescence emitted at NanoLuc peak wavelength (∼450 nm); BRET, luminescence emitted at ∼525 nm as a result of BRET. The two rightmost panels are ratiometric BRET images of HeLa cells just before the addition of histamine (*t*=86s) and at the first peak of the Ca^++^ oscillation evoked by histamine (*t*=106 s; scale bar, 20 μm); the temporal pattern of the Ca^++^ oscillation of these cells is depicted in **c**. (**c**) HeLa cells transfected with CalfluxVTN responding to 10 μM histamine (Hist(+), black trace) or vehicle (Hist(−), red trace). Arrow shows when histamine or vehicle was added. See also Supplementary Movie 1. The calibration curve generated for microscope analysis (Supplementary Fig. 8) is relevant to these data. (**d**) Quantification of the data depicted in **c** (For Hist+, *n*=15 cells from four separate experiments, for Hist-, *n*=14 cells from four separate experiments, mean±s.e.m., for the Hist+ versus Hist- comparison *P<0.05, paired *t*-test).

CalfluxVTN can be expressed in cultured cells under the control of the CAG promoter (Fig. 3a,b). In unperturbed, transfected HEK293 cells treated with the furimazine substrate^24^ the BRET ratio of CalfluxVTN is stable for at least one hour (Supplementary Fig. 3). Ca^++^ levels oscillate in HeLa cells after stimulation with histamine^27^,^28^,^29^, and CalfluxVTN faithfully reports this cycle in CalfluxVTN-transfected HeLa cells exposed to 10 μM histamine, as indicated by the oscillation of BRET ratio (Fig. 3c,d and Supplementary Movie 1). The data of Fig. 3 were derived from HeLa cells in serum-free medium, but CalfluxVTN and furimazine are equivalently useful in serum-containing medium, for example, when serum (FBS) is included in the medium, many cycles of Ca^++^ oscillation can also be measured by CalfluxVTN (Supplementary Fig. 4).

**Figure 4:**
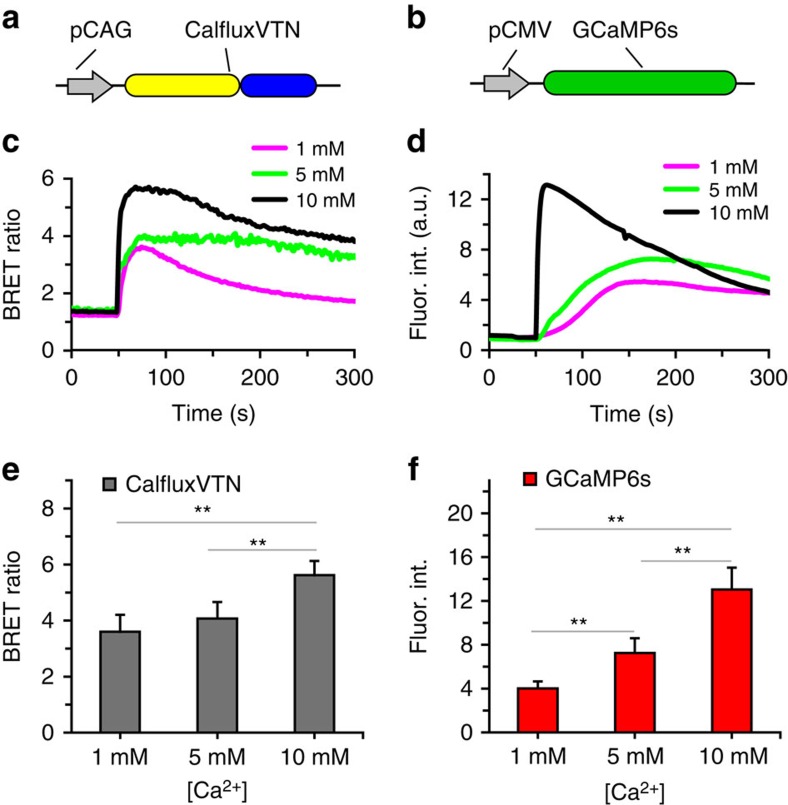
Comparison of the Ca^++^- induced responses between CalfluxVTN and GCaMP6s. (**a**) CalfluxVTN driven by pCAG and (**b**) GCaMP6s driven by pCMV in HEK293 cells. (**c**,**d**) In all, 10 μM ionomycin was added (at ∼50 s) to HEK293 cells in 1 mM, 5 mM or 10 mM CaCl_2_-containing medium expressing CalfluxVTN (**c**) or GCaMP6s (**d**). Plots depict the average responses. (**e**,**f**) Graphs showing the average peaks of the [Ca^++^]-dependent responses of CalfluxVTN (**e**) and GCaMP6s (**f**) to ionomycin induced Ca^++^—influx. (for each sample group, data come from four separate experiments with a total cell number between 17 and 24; mean±s.e.m., **P<0.01, paired *t*-test). The calibration curve generated for microscope analysis (Supplementary Fig. 8) is relevant to these data. a.u., arbitrary unit.

We compared the Ca^++^-responsiveness of CalfluxVTN with that of a well characterized fluorescence sensor of Ca^++^ fluxes, GCaMP6s (ref. 8). HEK293 cells were exposed to the Ca^++^ ionophore ionomycin in the presence or absence of various concentrations of extracellular CaCl_2_ (1, 5 and 10 mM CaCl_2_). The cells were transfected with either the CalfluxVTN expression construct (Fig. 4a) or the GCaMP6s expression construct (Fig. 4b). As shown in Fig. 4c,d, both sensors responded to the ionomycin-stimulated Ca^++^ flux with large changes in BRET ratio (CalfluxVTN) or fluorescence intensity (GCaMP6), with the magnitude of the response proportionally dependent upon the extracellular CaCl_2_ concentration. The dynamic range and sensitivity of response was somewhat larger with GCaMP6s than with CalfluxVTN (Fig. 4e,f). Therefore, as compared with well-characterized fluorescence Ca^++^ indicators, CalfluxVTN is also a stable and specific sensor of Ca^++^ in unstimulated cells and in cells that have been stimulated by different Ca^++^-altering treatments (that is, histamine and ionomycin).

### CalfluxVTN is an optimal optogenetic partner with melanopsin

**Figure 5:**
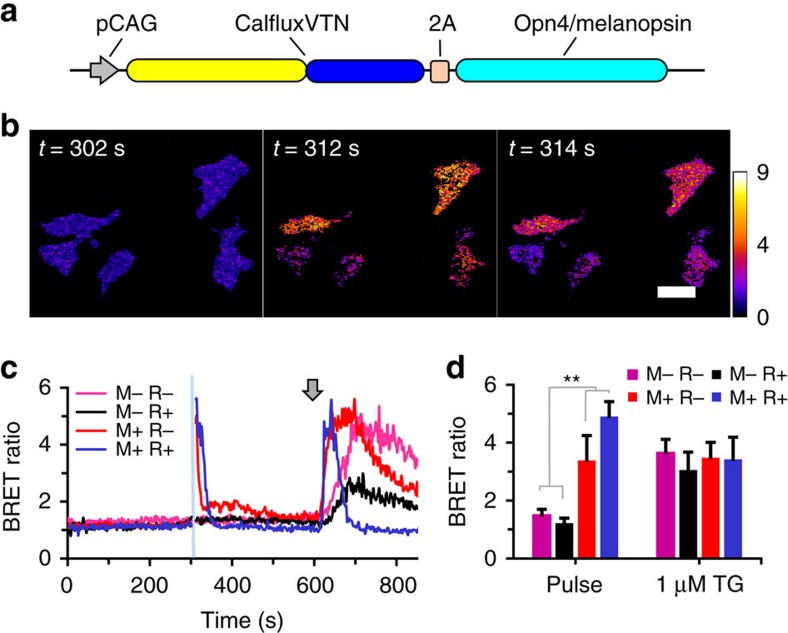
Coupling BRET Ca^++^ sensing with an optogenetics probe that regulates store-operated Ca^++^ (SOC) release. (**a**) Bicistronic pCAG-driven construct encoding CalfluxVTN and mouse melanopsin (*opn4*). (**b**) Photomicrographs of the ratiometric image of BRET emitted from HEK293 cells expressing CalfluxVTN and melanopsin (retinal-treated, M+/R+) in **c** over the time course just before (302 s) and for 2 s (times 312–314 s) after stimulation with a 10 s blue light pulse between 302–312 s (scale bar, 20 μm). (**c**) Optogenetically stimulated ratiometric changes in BRET after excitation of melanopsin with 10 s blue light (at blue line, time 302 s; pulse is 470 nm±30 nm) in HEK293 cells expressing either the construct in **a** or CalfluxVTN alone (M=transfected with *opn4*, R=treated with all-*trans*-retinal). HEK293 cells that do not express melanopsin (=M−, construct shown in Figs 3a and 4a) do not exhibit a BRET ratio change in response to the blue light pulse. Both groups of cells (M+ and M−) show large and persisting changes in BRET ratio in response to stimulation of SOC release by 1 μM thapsigargin (at arrow). Some of the preparations were additionally treated with 100 nM all-*trans*-retinal (R+) to reconstitute a larger amount of active melanopsin^4^. See also Supplementary Movie 2. (**d**) Quantification of the HEK293 cells’ responses in **c** of the peak BRET ratio after light stimulus (Pulse); peak ratio after thapsigargin was added (1 μM TG). (For each sample group, data come from three separate experiments with a total cell number between 14 and 16; mean±s.e.m., **P<0.01, *F*=20.7, M+ compared with M−, 2-factor analysis of variance (ANOVA)) The calibration curve generated for microscope analysis (Supplementary Fig. 8) is relevant to these data. TG, 1 μM thapsigargin.

The photopigment melanopsin triggers the release of internal calcium stores in response to blue light^4^,^5^. We transfected CalfluxVTN in HEK293 cells with (Fig. 5a) or without (Figs 3a and 4a) a co-expressed *opn4* sequence that encodes melanopsin (Fig. 5b). Active melanopsin depends upon binding of retinal that is present at low levels in mammalian cells^4^,^5^,^30^, but an even greater proportion of active melanopsin can be reconstituted *in vivo* when cell cultures are treated with all-*trans*-retinal. The BRET signal of HEK293 cells transfected with the CalfluxVTN/*opn4* construct (Fig. 5a) responded dramatically to optogenetic stimulation by a 10 s blue light pulse (Fig. 5b–d, Supplementary Movie 2), indicating a significant increase of intracellular Ca^++^. Cells treated with retinal (R+) exhibited a slightly stronger response to the light pulse than untreated cells (R−), as expected from the larger pool of active melanopsin in cells treated with retinal. On the other hand, cells transfected with CalfluxVTN but not *opn4* (Figs 3a and 4a) did not respond to the blue light pulse regardless of treatment with retinal (Fig. 5c,d). However, all of the cell groups transfected with CalfluxVTN (with or without *opn4*) responded to pharmacological release of internal Ca^++^ stores elicited by 1 μM thapsigargin (Fig. 5c,d), indicating that in each group the CalfluxVTN sensor was present and responsive to changes of cytosolic Ca^++^ levels. Note that all the measurements shown in Fig. 5b–d were conducted in darkness except for the 10 s light pulse.

**Figure 6:**
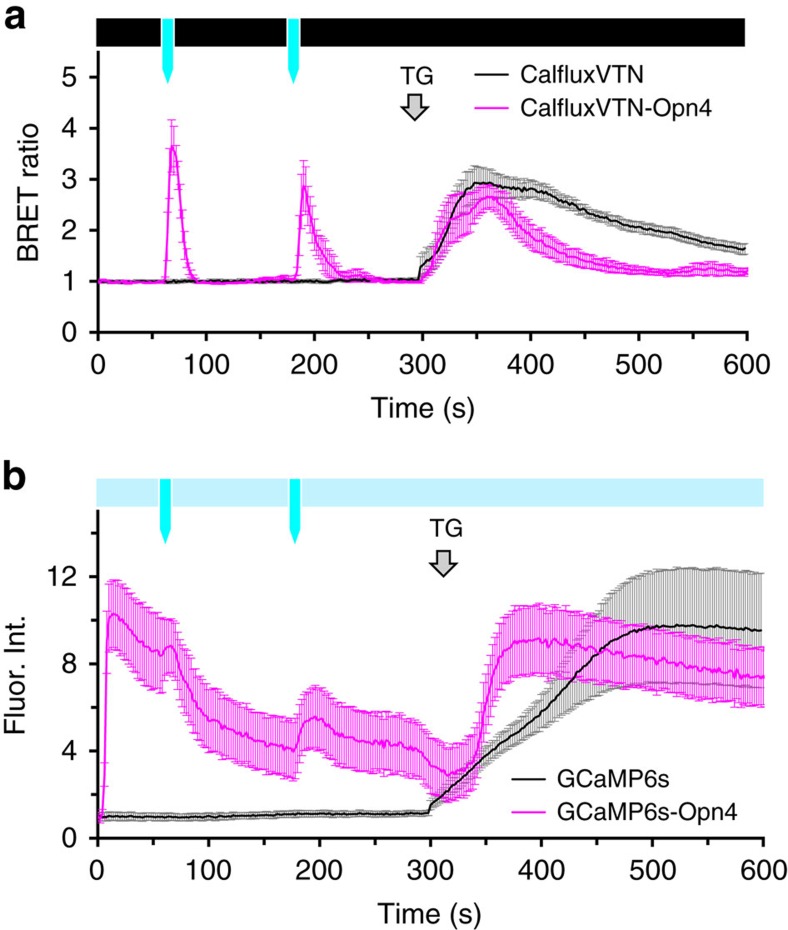
Comparison of coupling melanopsin-optogenetic stimulation of Ca^++^ influx with CalfluxVTN versus GCaMP6s. (**a**) CalfluxVTN and (**b**) GCaMP6s were expressed in HEK293 cells either alone or in a bicistronic vector that allows simultaneous expression of melanopsin (*opn4*). The average +/−s.e.m. responses of all the cells on one plate were plotted in response to blue light stimulation at 60 s and 180 s (indicated by the blue arrows). (**a**) For CalfluxVTN luminescence microscopy, the cells' responses were recorded in darkness (black bar above graph in **a**) and exposed to the two 1-second light pulses (blue arrows), (**b**) For GCamp6s fluorescence microscopy, dim excitation was maintained throughout to record GCamp6s fluorescence (light blue bar above graph in **b**). To stimulate melanopsin (Opn4), two 1-second blue light pulses were presented at 60 s and 180 s by increasing the light intensity of the fluorescence light source to an intensity that was equivalent to that used in **a** (bright blue arrows that punctuate the dim excitation indicated by the light blue bar). All light pulses exposed the entire field of cells. Grey arrow shows when thapsigargin (TG) was added to increase cytosolic Ca^++^. See Supplementary Fig. 5 for responses of individual cells. (For the data in this figure *n*=11 cells for CalfluxVTN, *n*=6 for CalfluxVTN-Opn4, *n*=7 cells for GCaMP6s and *n*=12 for GCaMP6s-Opn4; mean±s.e.m.) The calibration curve generated for microscope analysis (Supplementary Fig. 8) is relevant to these data.

As compared with a fluorescent Ca^++^-sensor, a BRET Ca^++^-sensor can avoid undesirable stimulation of a coupled optogenetic probe because the recording of Ca^++^-fluxes by a luminescent sensor is performed in darkness. Figure 6 depicts this advantage in a comparison of melanopsin-stimulated Ca^++^-fluxes by CalfluxVTN versus GCaMP6s. As also shown in Fig. 5, light pulses to the CalfluxVTN-Opn4-transfected HEK293 cells evoked large changes in BRET ratio, indicative of transient increases of cytosolic Ca^++^ (Fig. 6a). Except for the brief light pulses, the cells in Fig. 6a were otherwise in darkness. Moreover, thapsigargin also caused a large sustained change of cytosolic Ca^++^. Importantly, there was little variance in the responses among the cells (see error bars in Fig. 6a, and also the responses of individual cells in Supplementary Fig. 5a–c). In striking contrast, the same protocol performed with the GCaMP6s-Opn4 sensor exhibited significantly more heterogeneous responses, as indicated by the error bars in Fig. 6b (and also the responses of individual cells in Supplementary Fig. 5e,f). The GCaMP6s-transfected cells were continuously exposed to a dim excitation light to enable recording of the fluorescence of the sensor. The response of some cells transfected with GCaMP6s-Opn4 was very similar to that of the CalfluxVTN-Opn4-transfected cells (compare panel b with panel e in Supplementary Fig. 5), but there is considerable variability among the GCaMP6s-Opn4-transfected cells that is not present among the CalfluxVTN-Opn4-transfected cells. Because the variability of the response among the cells transfected with GCaMP6s without Opn4 is low (Fig. 6b and Supplementary Fig. 5d, first 300 s), our interpretation of this difference is that the dim excitation light quickly sparks some Ca^++^-flux in the GCaMP6s-Opn4-transfected cells, as indicated by the recordings in the first 12 s (Supplementary Fig. 5f). Therefore, the heterogeneity of these unintended responses is likely due to variability of expression level of melanopsin among the GCaMP6s-Opn4-transfected cells, whereas cells transfected with GCaMP6s (without Opn4) are not light-responsive.

### Calflux reports Ca^++^ flux in hippocampal neurons and slices

**Figure 7:**
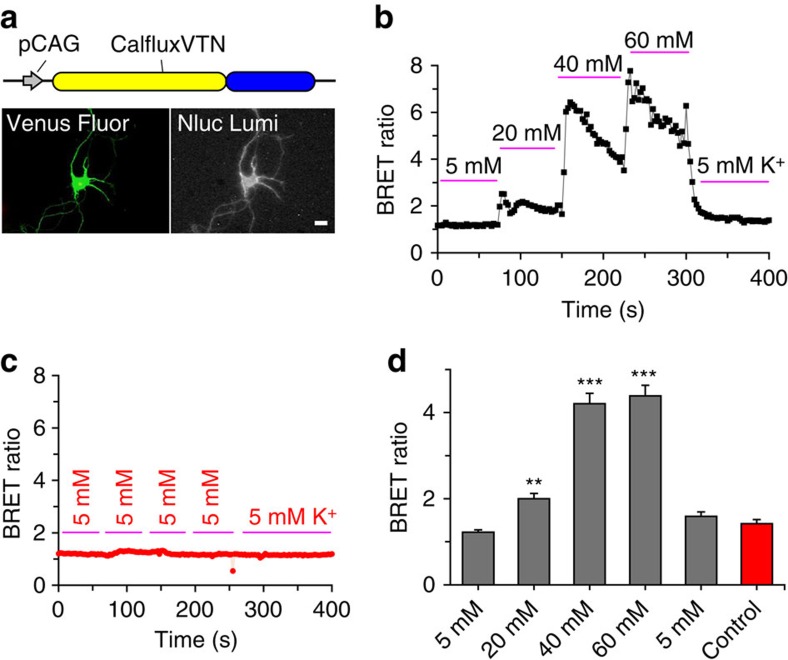
CalfluxVTN reports depolarization-evoked Ca^++^ fluxes in rat hippocampal neurons. (**a**) CalfluxVTN construct driven by CAG promoter (pCAG^37^) for mammalian cell expression (upper panel), and photomicrograph of a single hippocampal neuron expressing the construct. Fluorescence of the Venus moiety (lower left panel) and luminescence of NanoLuc (lower right panel) are shown (scale bar, 10 μm). (**b**) BRET ratio increased (indicating increase of intracellular [Ca^++^]) in hippocampal neurons as the medium was exchanged with increasing concentrations of K^+^ (from 5 to 60 mM KCl; *n*=6 independent cells). (**c**) When medium was exchanged repeatedly with control medium (5 mM KCl; *n*=3 independent cells), there was no change in BRET ratio, indicating that the changes observed in **b** were not due to physical manipulations. (**d**) Quantification of the neurons' responses to varying [K^+^] illustrated in **b** and **c** (mean±s.e.m. **P<0.01, ***P<0.001, 1-factor analysis of variance (ANOVA)). The calibration curve generated for microscope analysis (Supplementary Fig. 8) is relevant to these data.

Neuronal Ca^++^ levels increase in response to membrane depolarizations that open voltage-gated Ca^++^ channels^31^. Channel-opening depolarization conditions include neural activity (for example, action potentials) and high extracellular concentrations of K^+^ (ref. 31). To test whether CalfluxVTN could detect depolarization-induced Ca^++^ fluxes in mammalian neurons, dissociated rat hippocampal neurons at days 5–6 in culture were transfected using a modified calcium phosphate method^32^,^33^ with the CalfluxVTN-encoding vector (Fig. 7a). Approximately 4–5 days later, CalfluxVTN-expressing neurons (about 10% of the total population) could be detected by the fluorescence of the Venus moiety; these fluorescent cells also expressed robust NanoLuc luminescence in darkness (Fig. 7a). The BRET ratio of CalfluxVTN increased as a function of [K^+^] in the medium (Fig. 7b). These changes were reversible; when the high concentrations of K^+^ (for example, 40–60 mM KCl) were returned to the standard 5 mM KCl concentration, the BRET ratio returned to its original value (Fig. 7b,d). These changes in BRET ratio were not the result of physical manipulations or medium changes, because the same protocol of medium replacement using the standard 5 mM KCl medium for each change did not alter CalfluxVTN’s BRET ratio (Fig. 7c). Therefore, depolarizations elicited by extracellular K^+^ cause Ca^++^ fluxes that modulate CalfluxVTN’s BRET signal in a [K^+^]-dependent relationship, and these alterations are reversible.

**Figure 8:**
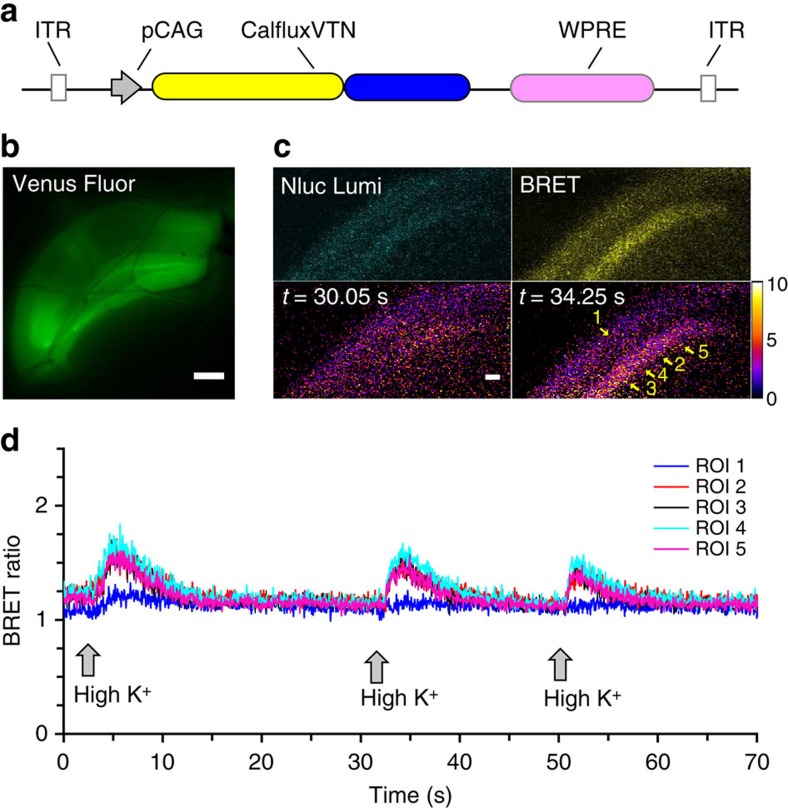
CalfluxVTN reports high K^+^ induced Ca^++^-flux in acute hippocampal brain slices. (**a**) Adeno-associated virus (AAV) vector-expressing CalfluxVTN, ITR, inverted terminal repeat of AAV, WPRE, regulatory element that stimulates expression of the insert. (**b**) Venus fluorescence signal of CalfluxVTN from acute brain slices containing dorsal hippocampus from mice injected with AAV-CalfluxVTN (scale bar, 300 um). (**c**) Top-left panel: image of NanoLuc emission signal (‘Nluc Lumi’) from hippocampal CA1/pyramidal layer slice, top-right panel: BRET signal of identical region in ‘Nluc Lumi’ panel (‘BRET’), lower panels: ratiometric image of same region, before (lower-left panel) and after (lower-right panel) high K^+^ stimulation (scale bar, 50 um). See also Supplementary Movie 3. (**d**) Results of a flow-through experiment where high K^+^ (80 mM) was applied three times sequentially to an acute hippocampal slice while recording the CalfluxVTN BRET ratio (indicating Ca^++^ influx). ROI’s represent the numbered regions of interest in **c** (lower-right panel). See Supplementary Fig. 6 for another example of hippocampal slice responses to high K^+^ stimulated depolarization. The calibration curve generated for microscope analysis (Supplementary Fig. 8) is relevant to these data.

To determine if the BRET Ca^++^ sensor could be used with brain slices, we developed an adeno-associated virus construct that expresses CalfluxVTN from the pCAG promoter (=AAV-CalfluxVTN, Fig. 8a). This AAV vector was stereotactically injected into the dorsal hippocampus of mice. Three weeks later, coronal brain slices containing the hippocampus were prepared and mounted in a flow-through chamber for imaging. The AAV vector transduced hippocampal cells, as confirmed by fluorescence of the Venus moiety of CalfluxVTN (Fig. 8b). Furimazine substrate was added to the slowly flowing oxygenated artificial cerebrospinal fluid (ACSF), and NanoLuc luminescence was easily visualized as well as BRET signal emanating from the stratum pyramidale of the CA1 region of the hippocampus (Fig. 8c). The ACSF was changed from 5 mM KCl to 80 mM KCl and back again three times. Each time the slice was exposed to the high K^+^ medium, the BRET ratio increased reversibly in the neuron cell body-dense stratum pyramidale of the CA1 region of the hippocampus (regions of interest (ROIs) 2–5, Fig. 8c,d, Supplementary Movie 3) but not in other regions (ROI 1, Fig. 8c,d). Another example of hippocampal Ca^++^ responses to K^+^-stimulated depolarizations is shown in Supplementary Fig. 6.

### Neuronal responses to optogenetically induced depolarization

**Figure 9:**
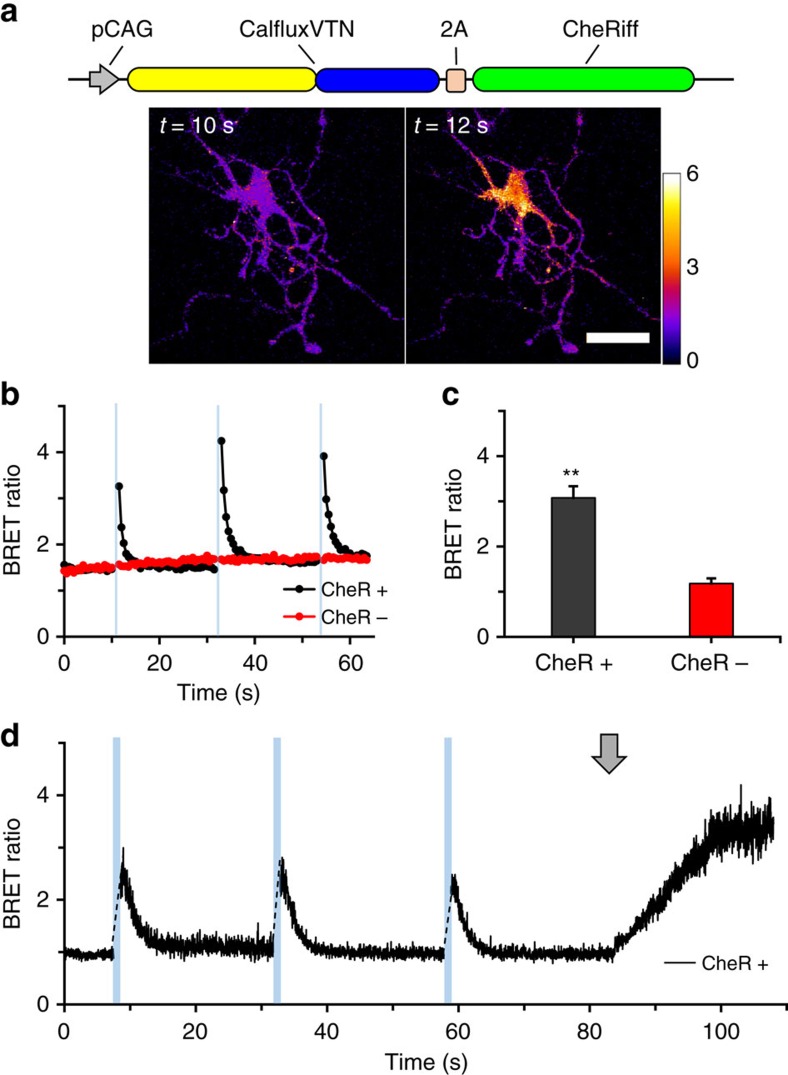
CalfluxVTN detects Ca^++^ fluxes elicited by optogenetically stimulated depolarization. (**a**) CheRiff is a ChR2 variant that evokes a peak photocurrent when excited by blue (460 nm) light^10^. CheRiff was co-expressed with CalfluxVTN in rat hippocampal neurons and emission wavelengths detected by light microscopy. Micrographs show BRET ratiometric images before (10 s) and after (12 s) blue light stimulation (scale bar, 20 μm). BRET ratio pseudocolour scale is shown next to the right micrograph. (**b**) One second blue light (470 nm±30 nm) pulses to cells expressing CalfluxVTN with (CheR+) and without (CheR-) CheRiff; both populations of cells were treated with 10 μM furimazine^24^ (imaged at 1 Hz recording rate). See also Supplementary Movie 4. (**c**) Quantification of the changes in BRET ratios seen in **b** after stimulation with blue light (with CheRiff, *n*=11 independent cells; without CheRiff, *n*=8 independent cells, mean±s.e.m. **P<0.01, *F*=34.6,1-factor analysis of variance (ANOVA)). (**d**) Responses of another hippocampal neuron transfected with CheRiff imaged at a higher recording speed (40 Hz). Blue bars indicate the time of 1-second blue light pulses, while the grey arrow indicates the addition of 80 mM high K^+^ medium. The calibration curve generated for microscope analysis (Supplementary Fig. 8) is relevant to these data. The neurons in **a**−**c** were transfected with DNA-Ca_3_(PO_4_)_2_ precipitation^32^,^33^,^38^, whereas the neurons in the experiment of **d** were transduced with the AAV-CalfluxVTN vector.

Exposure of neurons-expressing channelrhodopsin to blue light triggers excitatory cation fluxes that depolarize the plasma membrane, eliciting Ca^++^ fluxes into the neurons^2^,^3^. We transfected rat hippocampal neurons with a construct that co-expresses CalfluxVTN and the ChR2 variant CheRiff^10^ (Fig. 9a). As in the case of HeLa and HEK293 cells, CalfluxVTN-transfected hippocampal neurons emit NanoLuc luminescence and BRET (Fig. 9a). Exposure to 1 s pulses of blue light to neurons in darkness provoked a dramatic transient increase in BRET ratio of the luminescence measured immediately upon return to darkness (Fig. 9b,c, black trace), indicating that light stimulation of CheRiff elicits membrane depolarization and subsequent Ca^++^ flux that is detected by CalfluxVTN. Neurons transfected with the CalfluxVTN construct without CheRiff but treated with the furimazine substrate did not exhibit any change of BRET ratio in response to blue light pulses (Fig. 9b,c, red trace). The light-stimulated responses of CheRiff-expressing neurons can be imaged as shown in Fig. 9a where the spatial changes of CalfluxVTN are indicated by pseudocolour encoding of BRET ratio (scale to the right of Fig. 9a, Supplementary Movie 4).

The brightness of NanoLuc enables recording rates that were not possible with the previous generation of dimmer luciferases. These faster recording rates allow higher temporal resolution of cellular events. Figure 9d illustrates an experiment in which the recording rate was 40 Hz. The timing of the responses to light pulses and high K^+^ medium are well defined. On the other hand, a potential concern about the brightness of NanoLuc is that it might be so bright that it stimulates channelrhodopsin directly. This concern is unfounded, as we determined by varying the concentration of the substrate furimazine to alter the intensity of luminescence. If the luminescence intensity were sufficient to stimulate channelrhodopsin directly and thereby elicit Ca^++^ fluxes, the BRET ratio would be expected to increase as furimazine concentrations are raised in ChR2-expressing neurons, but not in neurons that are not expressing ChR2. Our measurements show that the BRET ratio is essentially constant in hippocampal neurons within a range of furimazine concentrations above and below our standard experimental condition that vary the intensity of luminescence emission (Supplementary Fig. 7). A constant BRET ratio was observed whether or not channelrhodopsin (the CheRiff variant) was expressed in the neurons (Supplementary Fig. 7).

## Discussion

As we proposed in 2012 (ref. 23), the characteristics of BRET provide an untapped opportunity to develop genetically encodable and ratiometric optical sensors that could be ideal for optogenetic applications, especially to prevent the cross-excitation that can occur between a fluorescent sensor and the optogenetic probe (for example, ChR2 or melanopsin). A sensor that requires continuous photonic excitation will likely stimulate the optogenetic probe to some extent, thereby perturbing the very process that the sensor is supposed to be non-invasively measuring. Simultaneously, a luminescence BRET sensor avoids other problems associated with fluorescence irradiation such as tissue autofluorescence, poor tissue penetration, intrinsic tissue photoresponsiveness (for example, retina or plant tissue) and excitation-induced tissue photodamage^15^,^21^,^22^,^23^. Moreover, while two-photon technology may reduce some of these problems (for example, autofluorescence and probe crosstalk), the high-order photon flux required for two-photon excitation can photobleach the probe and/or photodamage the tissue^14^. As we show in Figs 5 and 9, a BRET-based sensor can monitor a cellular parameter (for example, Ca^++^) in darkness, then a brief flash of light can be applied to stimulate the optogenetic probe, after which time the BRET sensor is consulted in darkness to assess the cellular response.

No technique is perfect, but the traditional liabilities of BRET/luminescence sensors have been greatly ameliorated by the newly developed luciferase NanoLuc^24^. In the past, BRET/luminescence assays have been hampered by the dimness of the luminescence level and the instability of the substrate needed for the luciferase-catalysed reaction (esp. coelenterazine). In both of these respects, however, NanoLuc is revolutionary^24^. NanoLuc is 100–150 × brighter than *Renilla* luciferase (RLuc) or firefly luciferase (FLuc)^24^. Even though it has been possible to enhance the brightness of RLuc by imaginative BRET partners^19^, RLuc remains much dimmer than NanoLuc, and consequently CalfluxVTN is 30–50 × brighter than the brightest RLuc-based Ca^++^ sensor, Nano-lantern(Ca^2+^)^19^,^34^. An ongoing advantage of luminescence over fluorescence techniques is its superior signal to noise ratio (SNR), and with NanoLuc's brightness, SNR is even better than with previously available luciferases. Moreover, the development of NanoLuc concomitantly entailed the evolution of a new substrate, furimazine, which produces less autoluminescence as well as being more stable in cell culture media that includes serum (as in Supplementary Fig. 4) than its ancestral substrate coelenterazine^24^. We confirmed that the luminescence level of NanoLuc expressed within cell cultures was strong and the BRET ratio stable for over one hour (Supplementary Fig. 3). Therefore, furimazine-based NanoLuc Ca^++^ sensors (such as CalfluxVTN) will be more manageable and stable than Ca^++^ BRET sensors such as BRAC or Nano-lantern(Ca^2+^) that use coelenterazine^19^,^34^. Nevertheless, furimazine is not as stable as some other bioluminescence substrates such as beetle luciferin, and consequently for long-term experiments (for example, circadian rhythm experiments), further optimization of substrate stability would be invaluable.

CalfluxVTN effectively tracks Ca^++^ transients resulting from Ca^++^ oscillations (Fig. 3c,d, Supplementary Fig. 4), high K^+^-induced depolarization (Figs 7 and 8), and optogenetic stimulation of melanopsin (Figs 5c,d and 6a) or ChR2 (Fig. 9), however, further testing will be required to ascertain the kinetic limits of this version of the sensor in tracking very rapid events, such as the Ca^++^ transients that accompany action potentials. The dynamic range of CalfluxVTN is large (∼10–12, Figs 1c and 2, Supplementary Fig. 8, and Supplementary Table 1) and not only dramatically surpasses the dynamic range of previous BRET reporters based on RLuc (Fig. 2, refs 19, 34), but surprisingly eclipses the dynamic range of its fluorescent parent Twitch-3 (dynamic range ∼7, Supplementary Table 1, ref. 16). However, the dynamic range and sensitivity of response is larger with the sixth-generation fluorescence sensor GCaMP6s than with this first-generation BRET sensor CalfluxVTN (Fig. 4). Undoubtedly further optimization of CalfluxVTN or similar sensors is warranted. Nonetheless, even in its current form, the large dynamic range and brightness of CalfluxVTN is responsible for the excellent SNR that is obvious in the responses to optogenetic stimulation (Figs 5, 6 and 9). The desirable properties of CalfluxVTN indicate its readiness to be applied to other biological questions that involve Ca^++^ fluxes. These may include *in vivo* applications. For example, FLuc-based reporters have successfully monitored the expression of gene expression continuously from the brain of freely moving mice for up to 3 weeks in constant darkness with a fiber-optic^35^. NanoLuc-based sensors are substantially brighter than FLuc-based sensors and therefore the successful development of this new generation of BRET sensors heralds a surge of novel applications of luminescence technology that circumvents the technical problems of fluorescence.

## Methods

### Construction of plasmids

Each fusion protein was constructed by polymerase chain reaction (PCR) cloning using pVenus-N1 (Addgene ID# 61854) as the template DNA for the Venus moiety and pNL1.1 (Promega) as template DNA for the NanoLuc moiety^24^. The Troponin C (TnC) domain (from *Opsanus tau*) was synthesized by overlap PCR using sequence information from Addgene (ID#: 49532). The TnC domain was inserted between Venus (N-terminal) and NanoLuc (C-terminal) using linker sequences RMQDA and PLAEL (Supplementary Tables 2 and 3). Conformational changes of TnC in response to Ca^++^ concentration [Ca^++^] allow varying configurations between the Venus and NanoLuc to promote BRET. The CalfluxVTN sequence was cloned into plasmids pRSETB (Invitrogen) and pCAG (from VSFP Butterfly 1.2, Addgene #: 47978) for bacterial and mammalian expression, respectively. For adeno-associated viral (AAV) transduction, CalfluxVTN and CalfluxVTN-2A-CheRiff were cloned into AAV-pCAG, which was modified from AAV-pCAG-FLEX-tdTomato-WPRE (Addgene #: 51503) and the plasmid was shipped to ViGene Biosciences (Rockville, MD, USA) where they created a mix of serotype 1+2 AAV particles.

### Protein purification and *in vitro* experiments

The pRSETB plasmid harboring CalfluxVTN was transformed into BL21 *Escherichia coli* for expression and purification of the fusion protein. The His6-tagged CalfluxVTN protein was purified with a TALON Metal Affinity Co^++^ Resin. The BRET signal of the purified CalfluxVTN protein, in response to varying [Ca^++^], was measured in Ca^++^ buffers from Molecular Probes by Life Technologies using a QuantaMaster fluorescence spectrophotometer from Photon Technology International Inc. (with the fluorescence excitation turned off for BRET and luminescence measurements). The Molecular Probes buffers were: buffer 1=10 mM EGTA, 100 mM KCl, 30 mM MOPS, pH 7.2; buffer 2=10 mM CaEGTA, 100 mM KCl, 30 mM MOPS, pH 7.2. Buffers were mixed to produce the varying free Ca^++^ concentrations. For all experiments, the NanoLuc substrate, furimazine (2-furanylmethyl-deoxy-coelenterazine), was added to a final concentration of 10 μM. For QuantaMaster measurements, ratiometric values were calculated by dividing the light emitted at the Venus peak (∼525 nm) by that emitted at the NanoLuc peak (∼450 nm), after a full scan of the light emission spectrum from 400 to 600 nm where every second nm was assayed.

### Comparison of CalfluxVTN versus GCaMP6s responses

CalfluxVTN and GCaMP6s (ref. 8) were expressed separately in HEK293 cells, and the cells were incubated in Ca^++^ buffers of varying concentrations (1 mM, 5 mM or 10 mM CaCl_2_; other components in mM: 0.49 MgCl_2_, 0.41 MgSO_4_, 5 KCl, 0.44 KH_2_PO_4_, 4.16 NaHCO_3_, 150 NaCl, 0.34 Na_2_HPO_4_, 10 HEPES (pH 7.2), and 0.6 % wt/vol D-glucose). The cells were then imaged on the microscope, and their responses to 10 μM ionomycin were recorded using either luminescence microscopy (CalfluxVTN) or epi-florescence microscopy (GCaMP6s).

### Coupling CalfluxVTN and GCaMP6s with optogenetic rhodopsins

Two bi-cistronic expression plasmid was created with CalfluxVTN plus mouse melanopsin (*opn4*) under the control of pCAG, and GCaMP6s plus *opn4* under the control of pCAG. The Ca^++^-sensor and Opn4 sequences were separated by the T2A linker^36^. For CalfluxVTN, another bi-cistronic expression vector was created in which the *opn4* sequence was replaced with the novel channelrhodopsin CheRiff^10^. Cells were transfected with these CalfluxVTN and GCaMP6s constructs (Figs 3a, 4a, 5a and 6; transfection as described in cellular expression and characterization) and stimulated with blue light pulses (470 nm±30 nm) of the durations specified in the relevant figure legends.

### Cellular expression and characterization

CalfluxVTN-containing plasmids in which the CalfluxVTN sequence was expressed under the control of the CAG promoter (pCAG^37^) were transfected via Lipofectamine 2,000 (ThermoFisher Scientific Inc.) or GeneCellin (BioCellChallenge) *in vitro* transfection reagents into either HEK293 or HeLa cells grown in DMEM media (Gibco). Approximately 48–96 h later, BRET responses were imaged as described below (Microscopy Imaging and Data Analysis). During real-time imaging, BRET ratio responsiveness to changes in cytosolic Ca^++^ was tested by adding 1 μM thapsigargin or 10 μM ionomycin to HEK cells, or by adding 10 μM histamine to HeLa cells.

Primary hippocampal neurons from prenatal rat fetuses were prepared and transfected via DNA-Ca_3_(PO_4_)_2_ precipitation^32^,^33^,^38^ with CalfluxVTN constructs under the control of the CAG promoter (pCAG^37^). Hippocampal neurons were also transduced via AAV particles (serotype AAV 1+2 mix). Pre-made AAV particles (ViGene Biosciences) were diluted in phosphate buffered saline (PBS; Gibco) and added to neurons after 1 week in culture. Five days later, the neurons were used for imaging experiments. For BRET imaging, the B27-supplemented Neurobasal growth medium (Gibco) for the neurons was replaced with a simple medium composed of 134 mM NaCl, 5 mM KCl, 10 mM D-glucose, 2 mM CaCl_2_, 1 mM MgCl_2_, 1 mM Na_2_HPO_4_, 20 mM HEPES pH 7.2. The neurons were depolarized by varying the concentration of K^+^ in the simple medium, which was achieved by reciprocally altering the concentrations of KCl and NaCl to maintain isosmoticity. Test media solutions of varying [K^+^] were exchanged in the imaging well by manual removal and replacement by syringes.

### Hippocampal viral injection and brain slice preparation

All animal experiments were approved by the Vanderbilt University Institutional Animal Care and Use Committee and were conducted according to that committee’s guidelines. On the day of surgery, male C57BL/6J mice were anesthetized with isoflurane and placed in a stereotaxic apparatus. A 33-gauge syringe needle was back filled with AAV-CalfluxVTN and 500 nl of the virus was injected bilaterally into the dorsal hippocampus at 50 nl min^−1^. Five minutes later, the syringe was withdrawn and the scalp wound was sutured closed. All animals remained healthy after the AAV injections. Three weeks after the stereotactic injections, hippocampal slices were collected from the mice. The mice were acclimated for 1 h in a sound-attenuating chamber, after which time they were anesthetized with isoflurane and decapitated. Brains were removed quickly and immediately transferred to a 1–4 °C oxygenated dissecting solution (in mM: 183 sucrose, 20 NaCl, 0.5 KCl, 2 CaCl_2_, 1 MgCl_2_, 1.4 NaH_2_PO_4_, 10 glucose, 26 NaHCO_3_, pH 7.2). Coronal brain slices (300 μm) containing the hippocampus were prepared with a vibratome (Leica Biosystems LLC). Slices were then transferred to a holding chamber for imaging that contained warm oxygenated (ACSF: 119 mM NaCl, 26.2 mM NaHCO_3_, 2.5 mM KCl, 1 mM NaH_2_PO_4_, 1.3 mM MgCl_2_, 10 mM D-glucose, 2.5 mM CaCl_2_, pH 7.2).

### Microscopic imaging

Cultured cells (HeLa or HEK293), primary neurons and acute tissue slices were imaged with an inverted Olympus IX-71 epi-fluorescence microscope inside a temperature-controlled, light-tight box with a cooled electron bombardment-CCD (charge-coupled device) camera (Hamamatsu Photonics C7190-13W) at a maximum frame rate of 4 Hz (ref. 22) (except for Fig. 9d, which was imaged at 40 Hz). To capture the BRET signals, blue and yellow filters were rotated in the emitting light path of the microscope. The blue filter (for NanoLuc luminescence) was a 480 nm±40 nm bandpass filter, and the yellow filter (or BRET to the Venus) was a 520 nm longpass filter. For faster imaging (for example, Fig. 9d at 40 Hz), an electron multiplying (EM)-CCD camera (Hamamatsu ImagEM X2) was used with comparable results. This EM-CCD camera was coupled to a light splitter (Hamamatsu W-View Gemini) that used a dichroic filter to separate the blue and yellow light and projected them onto distinct regions of the camera’s CCD chip. This allowed for simultaneous collection of blue and yellow wavelengths for optimal temporal resolution. During luminescence microscopy, all images were collected in complete darkness.

### Data analysis

The images were analysed with ImageJ software (NIH) using background-subtracted images collected from the blue and yellow channels. After background subtraction, a simple mathematical division of the yellow wavelength intensities by the blue wavelengths produced the BRET ratio values that are plotted throughout the article. The average light intensity of blue versus yellow from regions of interest within the cells was compared pixel by pixel to obtain ratiometric BRET estimates of cytosolic Ca^++^ over the image. To create the ratiometric photos and movies (for example, Fig. 5b), the backgrounds of both the blue and yellow channels were subtracted using the ImageJ background subtraction function. Then, the blue wavelength images were used as a template to create an 8-bit image that separated the cells completely from the background (cell region was given a value of 255, and background was given 0). The blue and yellow images were then multiplied by this template image where the background was held at 0 and areas within cells were given the full bit value. The resulting images were converted from 16-bit (from camera) to a 32-bit to mitigate losing data after the multiplication step. To finally achieve the ratiometric image, the yellow emission needed to be divided by the blue emission, but to prevent dividing by 0, a grey value of 1 (negligible, since it is out of 4.3 × 10^9^) was added to all the pixels in the blue images, so that dividing the yellow values by the blue values would not result in division by 0 at any pixel. When the final yellow images were divided by the final blue images, the ratio changes that are indicative of [Ca^++^] were observed (as indicated by the calibration curve in Fig. 1b,c or in Supplementary Fig. 8). The ImageJ lookup table (LUT) named ‘fire’ was applied to the images to better highlight the areas were [Ca^++^] was changing.

Note that the BRET ratio scale as a function of [Ca^++^](Ca^++^ buffers from Molecular Probes) ranges over 1.0–12.1 for the QuantaMaster *in vitro* measurements (Fig. 1b,c), but the same calibration curve generated with purified CalfluxVTN on the microscopic setup ranges over 1.0–10.5 (Supplementary Fig. 8). This difference is due to the BRET ratio from QuantaMaster measurements being calculated from peak values of the blue emission (∼450 nm) versus the peak values of the yellow emission (∼525 nm, Fig. 1b,c) of spectroscopic measurements, whereas the BRET ratio calculated from the microscopic setup includes all blue emission in the range of 440–520 nm versus all emission of wavelengths longer than 520 nm (Supplementary Fig. 8). Additionally, a higher concentration of CalfluxVTN protein was required for the microscopy calibration curve because the electron bombardment-CCD and EM-CCD cameras are less sensitive than the QuantaMaster. These differences caused a small difference in the BRET ratio range for any particular [Ca^++^]. Because the imaging data illustrated in Figs 3, 4, 5, 6, 7, 8, 9 (and Supplementary Figs 3–7) was generated from microscopic measurements, the BRET ratio calibration curve for ‘microscope’ depicted in Supplementary Fig. 8 should be used to evaluate those experiments, while the *in vitro* experiments with purified CalfluxVTN protein (Figs 1b and 2, Supplementary Figs 1 and 2, Supplementary Table 1) should be compared to the calibration curve in Fig. 1c (or the equivalent ‘QuantaMaster’ curve in Supplementary Fig. 8).

Statistical significance was tested using the Data Analysis Tool Pack from Microsoft Excel (Microsoft). The Hill coefficient and *K*_d_ values (Fig. 1c) were determined using OriginLab 6 software (OriginLab).

### Data availability

All relevant data are available from the authors, and the DNA construct encoding CalfluxVTN has been deposited with AddGene to make it freely available to other researchers.

## Additional information

**How to cite this article**: Yang, J. *et al*. Coupling optogenetic stimulation with NanoLuc-based luminescence (BRET) Ca^++^ sensing. *Nat. Commun.* **7**, 13268 doi: 10.1038/ncomms13268 (2016).

**Publisher's note:** Springer Nature remains neutral with regard to jurisdictional claims in published maps and institutional affiliations.

## Supplementary Material

Supplementary Information Supplementary Figures 1-8, Supplementary Tables 1-3, Supplementary Methods, Supplementary References.

Supplementary Movie 1 Cytosolic Ca++ changes in multiple HeLa cells expressing pCAGCalfluxVTN as stimulated by 10 μM histamine (40x microscopic objective). Ratiometric BRET images are shown. Histamine addition is indicated in the video when the text "Histamine" appears. The histamine is not washed out after its addition. These images were taken using the filter switching protocol (see Supplementary Methods). The acquisition speed for these images was 1 Hz.

Supplementary Movie 2 HEK293 cells expressing pCAG-CalfluxVTN-2A-Opn4 after light stimulation (gray flash) and store-operated Ca++ release from intracellular stores (via 1 μM thapsigargin)(40x microscopic objective). Ratiometric BRET images are shown. These images were taken using the filter switching protocol (see Supplementary Methods). The acquisition speed for these images was 0.5 Hz.

Supplementary Movie 3 Acute hippocampal slices expressing pCAG-CalfluxVTN responding to repeated bath applications of 80 mM K+ ACSF (10x microscopic objective). Ratiometric BRET images are shown. Eighty mM K+ addition is indicated in the video when the text "High K+" appears. Because these slices are exposed to a constant flow of ACSF, the high K+ ACSF is washed out after its addition by standard ACSF. The initial blue and yellow images were collected simultaneously were collected using the Hamamatsu Gemini W-View light splitter (see Supplementary Methods) so that the temporal resolution is optimized. Pixels were binned 2x2. The acquisition speed for these images was 20 Hz.

Supplementary Movie 4 Ca++ changes in primary hippocampal neurons expressing pCAGCalfluxVTN-2A-CheRiff after repeated light stimulation (grey flashes)(40x microscopic objective). Ratiometric BRET images are shown. These images were taken using the filter switching protocol (see Supplementary Methods). The acquisition speed for these images was 2 Hz.
