# Supplementary material for: Coupling optogenetic stimulation with NanoLuc-based luminescence (BRET) Ca++ sensing
Source: Nat Commun. 2016 Oct 27;7:13268. doi: 10.1038/ncomms13268 (PMC5476805; doi:10.1038/ncomms13268)
Supplement: Supplementary Information — Supplementary Figures 1-8, Supplementary Tables 1-3, Supplementary Methods, Supplementary References. [file ncomms13268-s1.pdf]

# Supplementary Figures

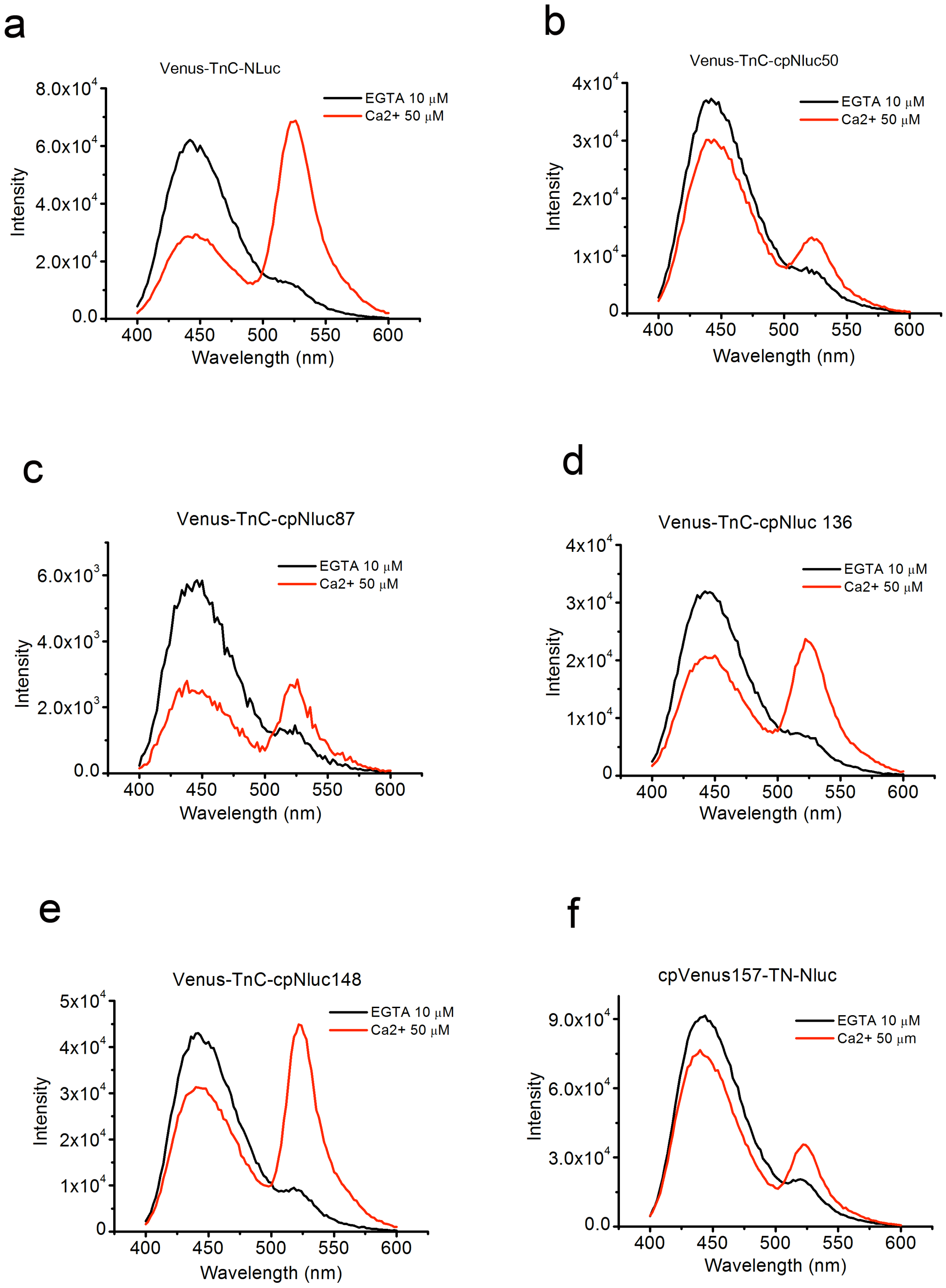

g

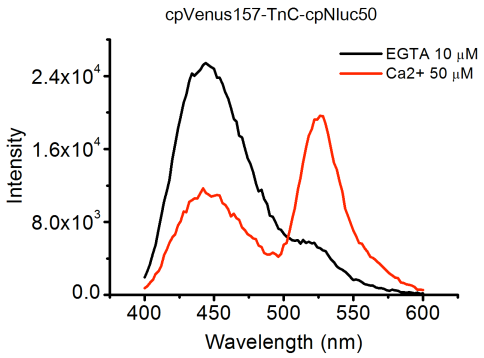
cpVenus157-TnC-cpNluc50
EGTA 10 μM
Ca2+ 50 μM
Intensity
2.4x10^4
1.6x10^4
8.0x10^3
0.0
400
450
500
550
600
Wavelength (nm)

h

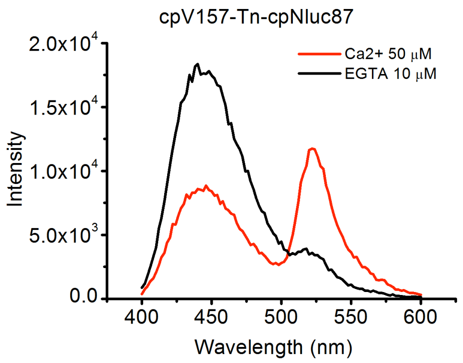
cpV157-Tn-cpNluc87
Ca2+ 50 μM
EGTA 10 μM
Intensity
2.0x10^4
1.5x10^4
1.0x10^4
5.0x10^3
0.0
400
450
500
550
600
Wavelength (nm)

i

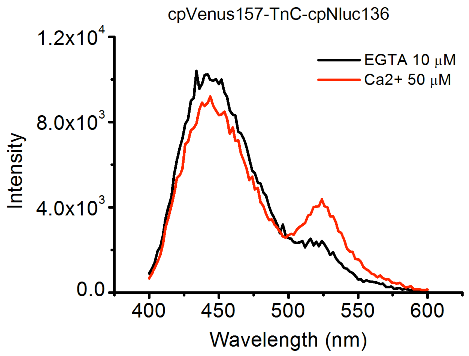
cpVenus157-TnC-cpNluc136
EGTA 10 μM
Ca2+ 50 μM
Intensity
1.2x10^4
8.0x10^3
4.0x10^3
0.0
400
450
500
550
600
Wavelength (nm)

j

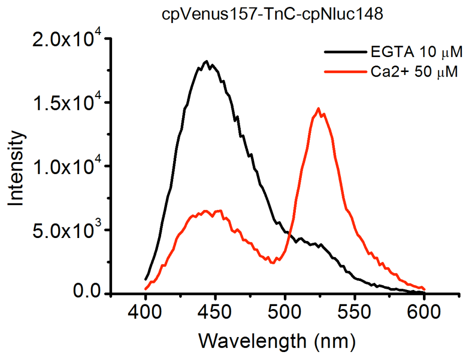
cpVenus157-TnC-cpNluc148
EGTA 10 μM
Ca2+ 50 μM
Intensity
2.0x10^4
1.5x10^4
1.0x10^4
5.0x10^3
0.0
400
450
500
550
600
Wavelength (nm)

k

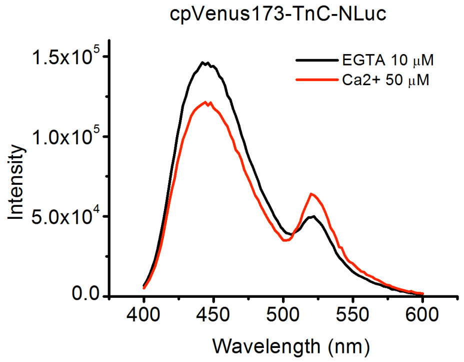
cpVenus173-TnC-NLuc
EGTA 10 μM
Ca2+ 50 μM
Intensity
1.5x10^5
1.0x10^5
5.0x10^4
0.0
400
450
500
550
600
Wavelength (nm)

l

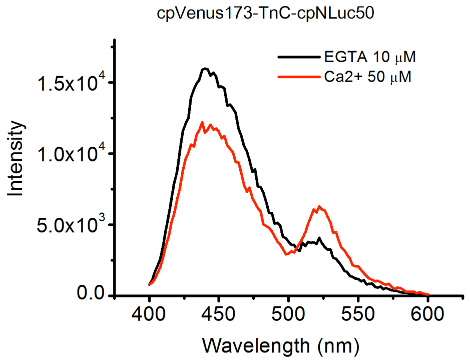
cpVenus173-TnC-cpNLuc50
EGTA 10 μM
Ca2+ 50 μM
Intensity
1.5x10^4
1.0x10^4
5.0x10^3
0.0
400
450
500
550
600
Wavelength (nm)

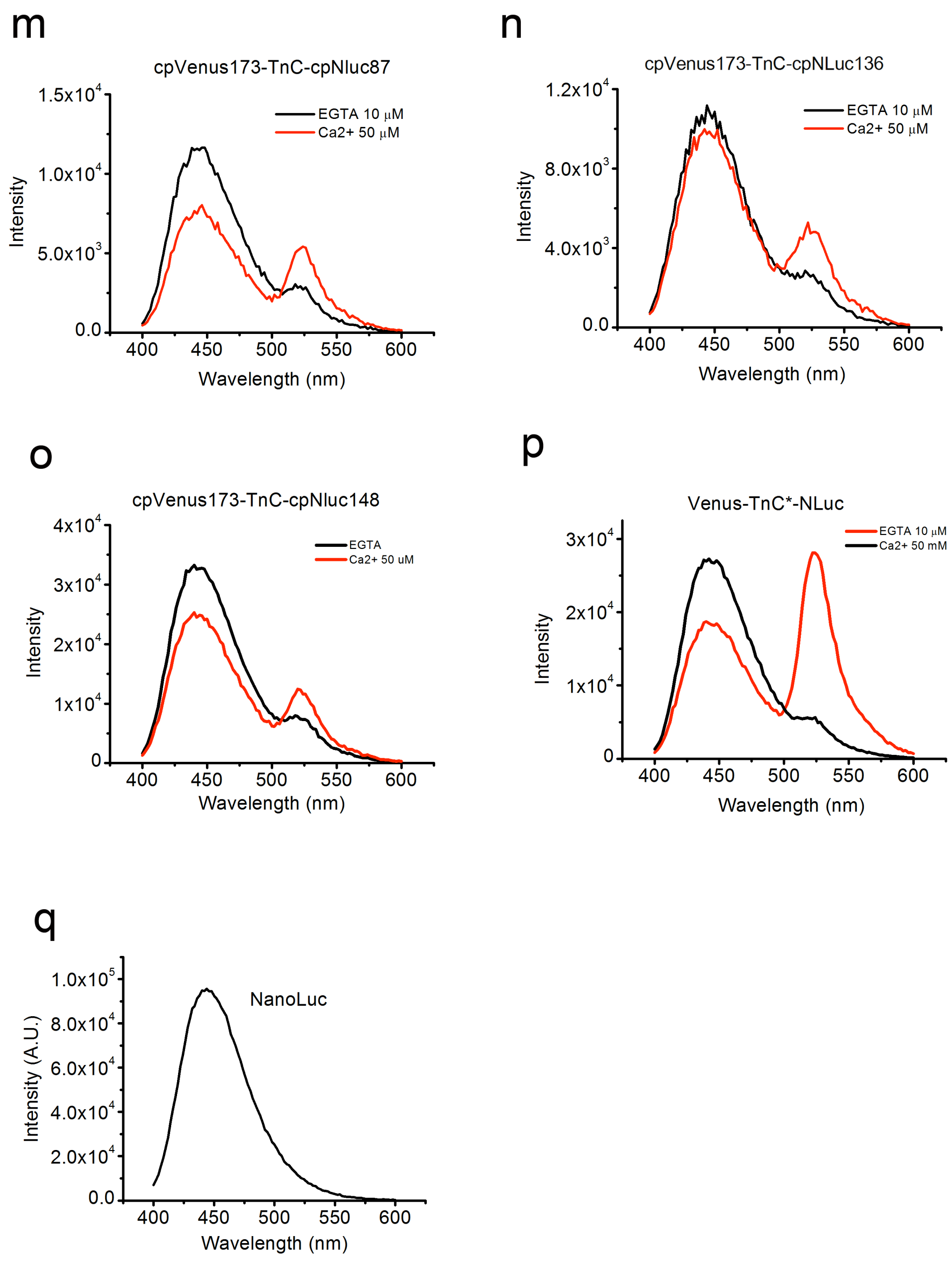


**Supplementary Fig. 1.** Characterization of the different $Ca^{++}$-sensitive BRET sensor iterations. **(a-o).** The troponin C domain (from Twitch-3) used in CalfluxVTN was cloned in a variety of combinations between the NanoLuc luciferase (NLuc, BRET donor) and Venus (BRET acceptor) and the response to high (50 μM $Ca^{++}$) and low (10 μM EGTA without added $CaCl_2$) $Ca^{++}$ is shown. The version shown in panel a is the selected version used in all experiments reported in this paper and called CalfluxVTN. **(p)** VTN 1.0 = Venus-TnC*-NLuc. The TnC* refers to the linkers used in the troponin domain from Twitch-2 (all the constructs in panels A-O have the TnC domain and linkers from Twitch-3). **(q)** Emission spectrum of NanoLuc luciferase alone.

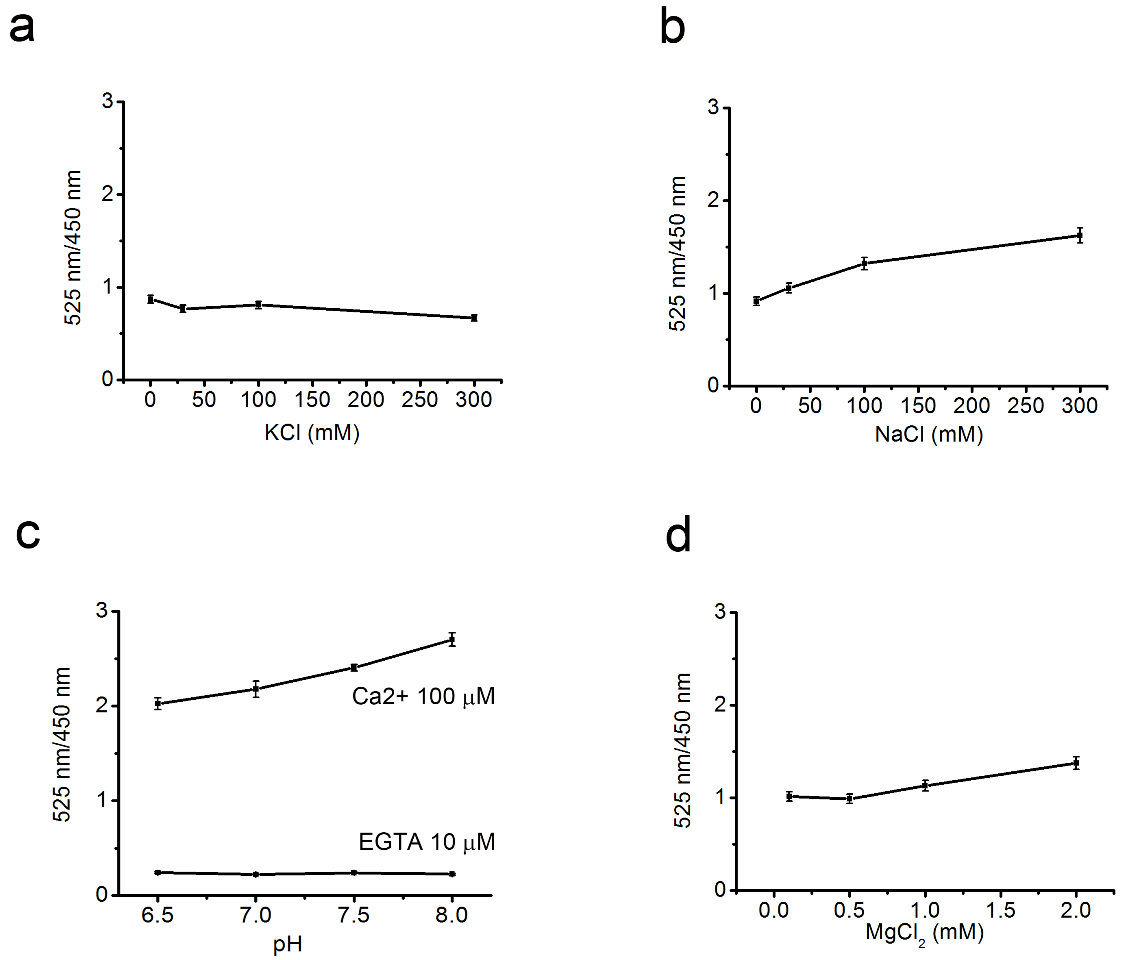


**Supplementary Fig. 2.** Insensitivity of CalfluxVTN to other ions *in vitro*. CalfluxVTN was purified via a His-6 tag and the BRET ratio was recorded in solutions with increasing concentrations of ions as shown. The BRET ratio in response to increasing [$K^+$] (panel **a**); [$Na^+$] (panel **b**); pH (panel **c**); [$Mg^{++}$] (panel **d)**. Data points represent mean ± S.D.

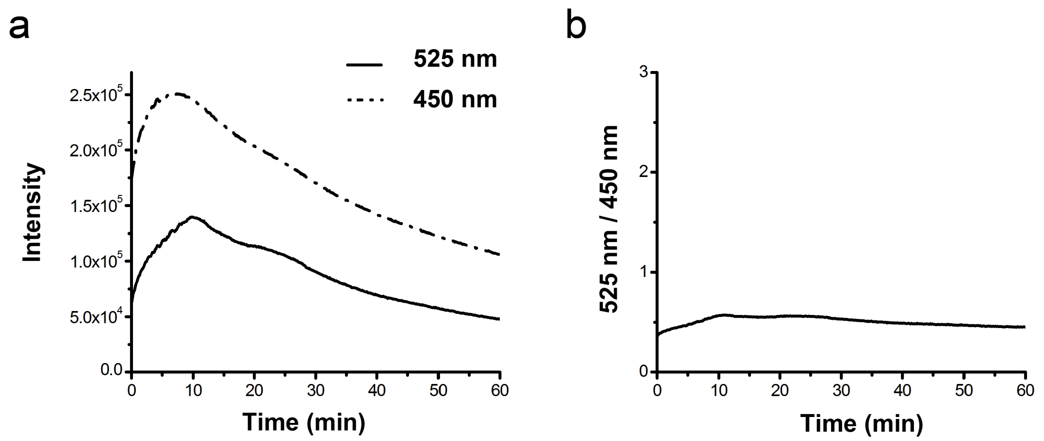


**Supplementary Fig. 3.** Stability of BRET signal for CalfluxVTN expressed in HEK293 cells. **(a)** HEK293 cells expressing CalfluxVTN were suspended in imaging media and the intensity of NanoLuc luminescence (NLuc) and the intensity of the resonance transfer to Venus (BRET) were monitored over time. **(b)** Stability of the BRET ratio over the course of 1 hour.

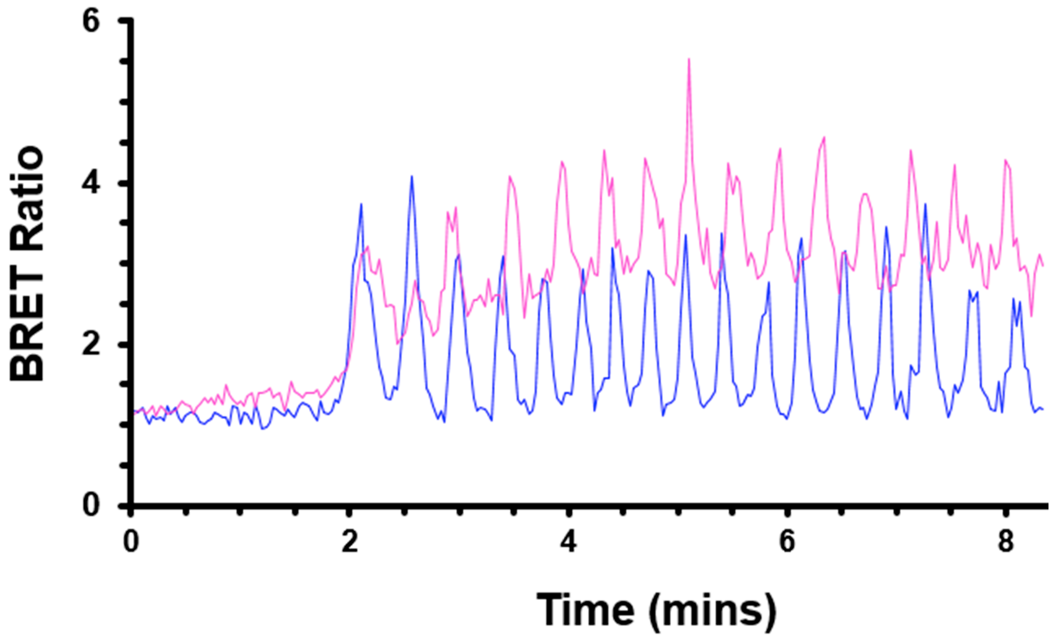


**Supplementary Fig. 4.** BRET ratio values collected in two different HeLa cells after 10 μM histamine was added to the medium at ~1.7 minutes. The medium consisted of FluoroBrite$^{TM}$ DMEM, 10 % fetal Bovine serum (FBS), and the experiment was carried out at 37ºC. Each trace (blue vs. pink) represents one HeLa cell's $Ca^{++}$ response to histamine stimulation.

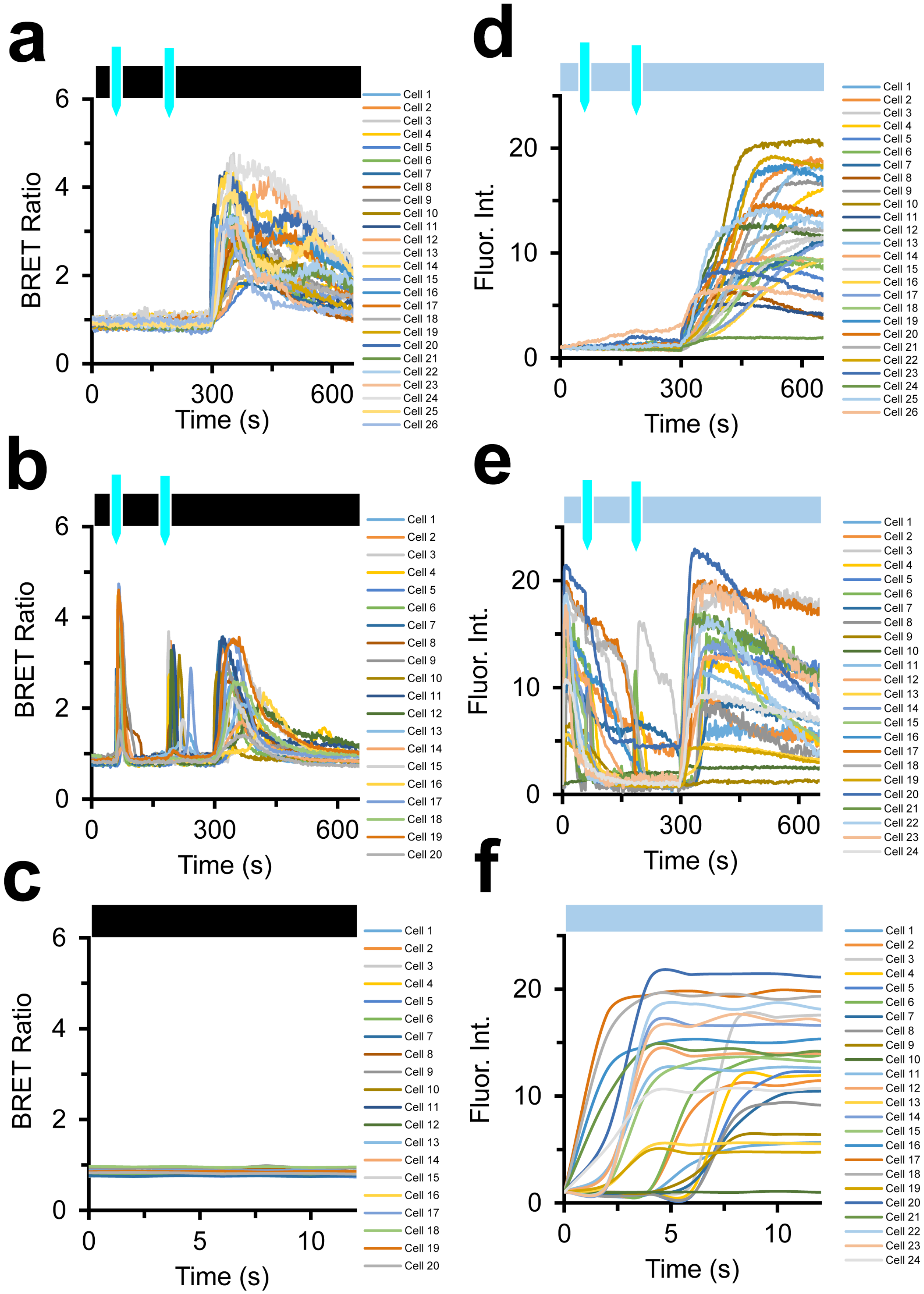
a
BRET Ratio
Time (s)
0
2
4
6
300
600
Cell 1
Cell 2
Cell 3
Cell 4
Cell 5
Cell 6
Cell 7
Cell 8
Cell 9
Cell 10
Cell 11
Cell 12
Cell 13
Cell 14
Cell 15
Cell 16
Cell 17
Cell 18
Cell 19
Cell 20
Cell 21
Cell 22
Cell 23
Cell 24
Cell 25
Cell 26
b
BRET Ratio
Time (s)
0
2
4
6
300
600
Cell 1
Cell 2
Cell 3
Cell 4
Cell 5
Cell 6
Cell 7
Cell 8
Cell 9
Cell 10
Cell 11
Cell 12
Cell 13
Cell 14
Cell 15
Cell 16
Cell 17
Cell 18
Cell 19
Cell 20
c
BRET Ratio
Time (s)
0
2
4
6
5
10
Cell 1
Cell 2
Cell 3
Cell 4
Cell 5
Cell 6
Cell 7
Cell 8
Cell 9
Cell 10
Cell 11
Cell 12
Cell 13
Cell 14
Cell 15
Cell 16
Cell 17
Cell 18
Cell 19
Cell 20
d
Fluor. Int.
Time (s)
0
10
20
300
600
Cell 1
Cell 2
Cell 3
Cell 4
Cell 5
Cell 6
Cell 7
Cell 8
Cell 9
Cell 10
Cell 11
Cell 12
Cell 13
Cell 14
Cell 15
Cell 16
Cell 17
Cell 18
Cell 19
Cell 20
Cell 21
Cell 22
Cell 23
Cell 24
Cell 25
Cell 26
e
Fluor. Int.
Time (s)
0
10
20
300
600
Cell 1
Cell 2
Cell 3
Cell 4
Cell 5
Cell 6
Cell 7
Cell 8
Cell 9
Cell 10
Cell 11
Cell 12
Cell 13
Cell 14
Cell 15
Cell 16
Cell 17
Cell 18
Cell 19
Cell 20
Cell 21
Cell 22
Cell 23
Cell 24
f
Fluor. Int.
Time (s)
0
10
20
5
10
Cell 1
Cell 2
Cell 3
Cell 4
Cell 5
Cell 6
Cell 7
Cell 8
Cell 9
Cell 10
Cell 11
Cell 12
Cell 13
Cell 14
Cell 15
Cell 16
Cell 17
Cell 18
Cell 19
Cell 20
Cell 21
Cell 22
Cell 23
Cell 24

**Supplementary Fig. 5.** Comparison of individual HEK293 cells used to test CalfluxVTN versus GCaMP6s as optimal optogenetic partners with melanopsin (Opn4). The CalfluxVTN alone **(a)**, and the CalfluxVTN-2A-Opn4 **(b and c)** were imaged in darkness with two, whole-field, blue light flashes at 60 s and 180 s (black bar with blue tick marks) while the blue light flashes in the GCaMP6s alone **(d)** and the GCaMP6s-2A-Opn4 **(e and f)** were generated by increasing the intensity of the excitation light from dim continuous background (to excite the fluorescence of GCaMP6s) to bright 1 sec pulses at 60 and 180 sec to stimulate melanopsin (same light intensity as the pulses given to the CalfluxVTN samples). Panel c is the same data as panel b and panel f is the same as panel e except that panels c and f depict the first 12 s of recording only. Gray arrows with black outlines depict where 1 μM Thapsigargin was added to the cells.

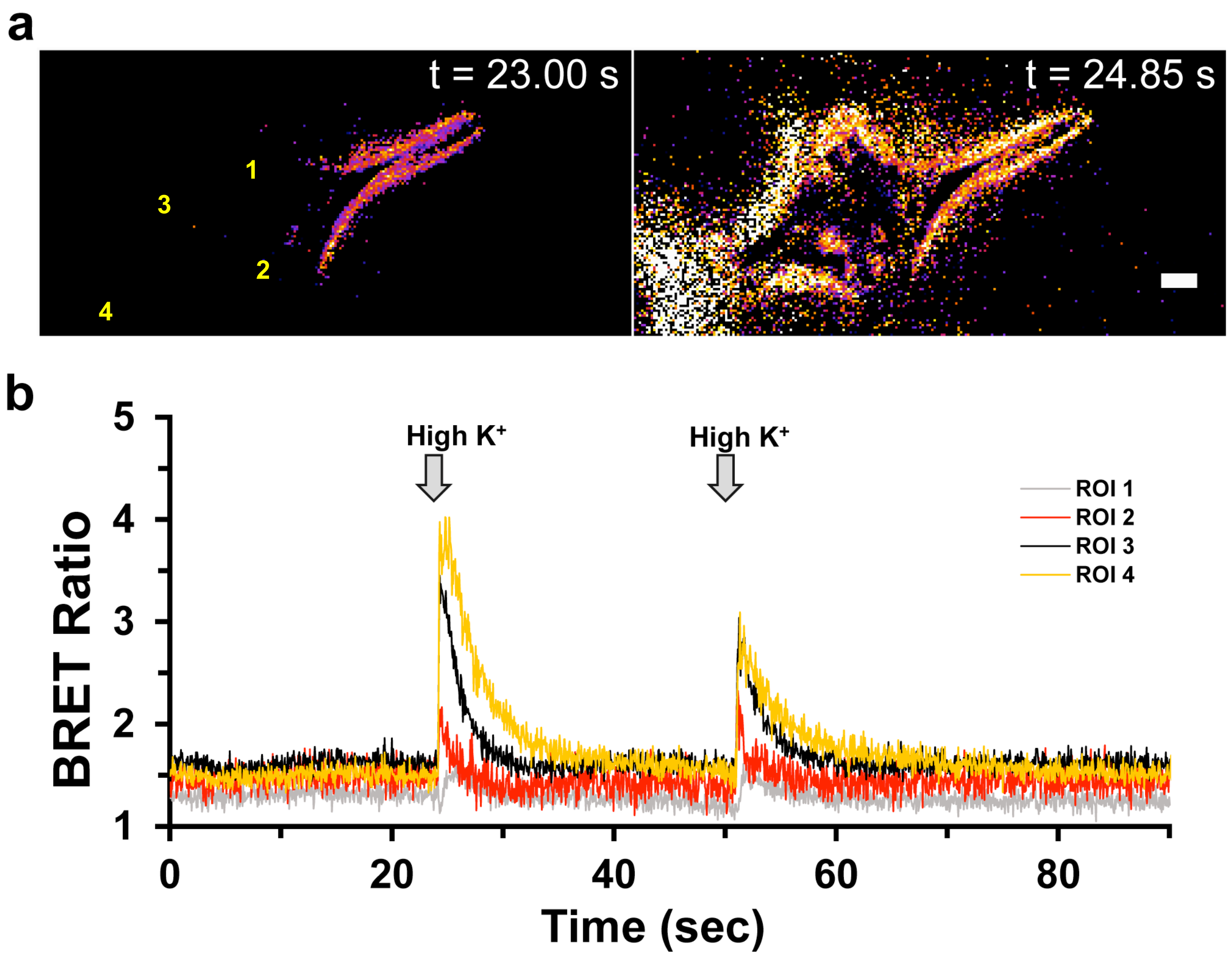


**Supplementary Fig. 6.** Acute, coronal brain slice containing the dorsal hippocampus shows $Ca^{++}$ transients resulting from high $K^{+}$ (80 mM) stimulation. (a) BRET ratio images just before (left) and just after (right) high $K^{+}$ addition to the flow apparatus. Numbers correspond to regions of interest (ROI) in panel b. (b) BRET ratio values come from multiple ROIs within the acute dorsal hippocampal slice responding to high $K^{+}$ (80 mM) stimulation with $Ca^{++}$ transients. Image acquisition speed was 20 Hz. (scale bar = 50 μm)

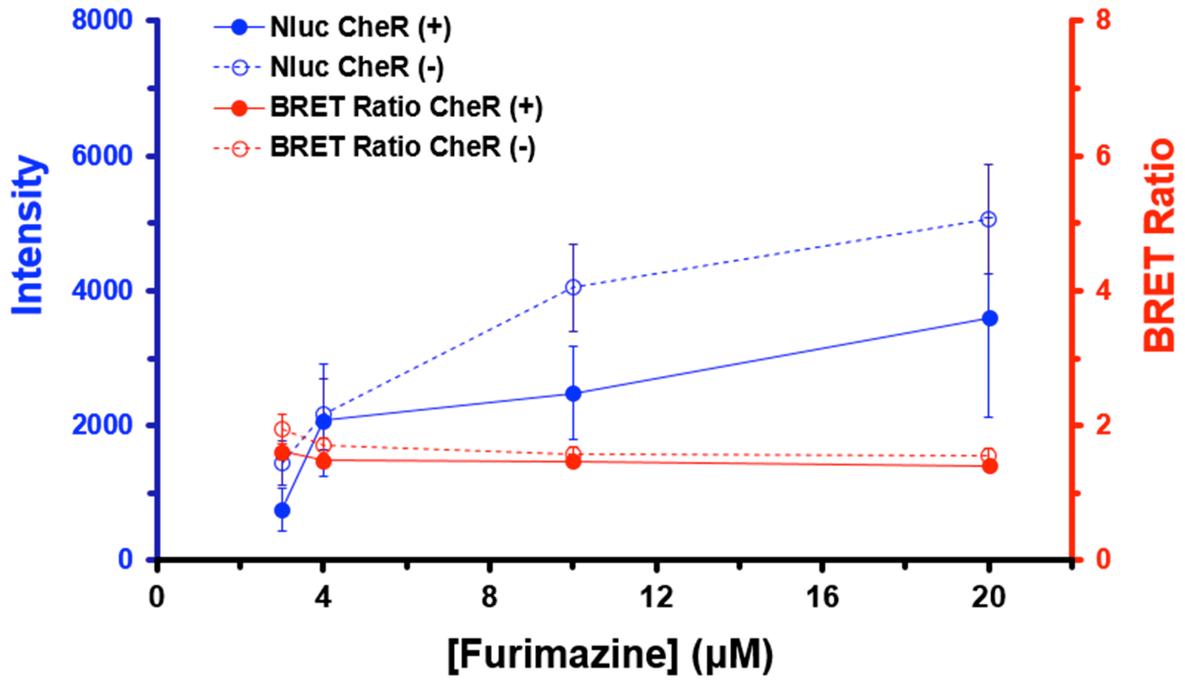


**Supplementary Fig. 7.** Comparison of the relationship between Nanoluc (Nluc) intensity and the corresponding BRET ratio in hippocampal neurons expressing CalfluxVTN and CheRiff (CheR) simultaneously. Furimazine concentration was increased in the same neurons (CalfluxVTN with and without CheRiff) and both the intensity of the Nluc signal and the BRET ratio were immediately measured. Luminescence intensity increased with increases of furimazine concentration, but the BRET ratio remained stable (mean ± S.E.M.). Ten micromolar furimazine is the standard concentration used in the experiments reported in this study.

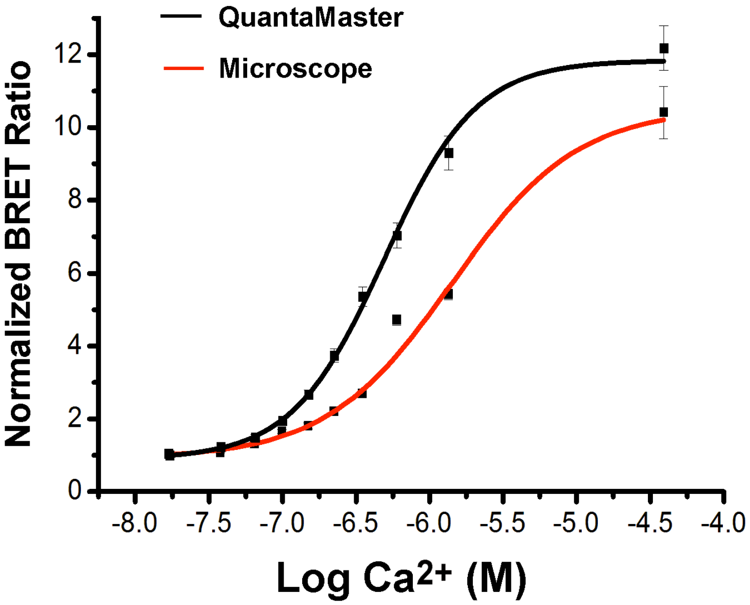


**Supplementary Fig. 8.** BRET ratio calibration curve generated as in Fig. 1c with purified CalfluxVTN and $Ca^{++}$ buffers from Molecular Probes® except that these $Ca^{++}$-buffered solutions were placed on a microscope slide (red trace) and imaged on the microscopic setup. In this case, the "Venus" signal is all the light emitted from 520 nm and longer, whereas the "NanoLuc" signal is all the light emitted in the range of 440-520 nm. The BRET ratio is calculated from those signals as "Venus"/"NanoLuc". For comparison, the calibration curve from Fig. 1c was included (black trace). Data points represent the mean ± S.E.M.

## Supplementary Tables

| | Acceptor | | | | |
|---|---|---|---|---|---|
| **Donors** | Venus | cpVenus157 | cpVenus173 | * Twitch-2 | * Twitch-3 |
| cpNluc50 | 1.15 | 6.77 | 1.10 | - | - |
| cpNLuc87 | 4.58 | 6.20 | 1.79 | - | - |
| cpNLuc136 | 4.65 | 1.10 | 1.00 | - | - |
| cpNLuc148 | 6.18 | 10.00 | 1.47 | - | - |
| NLuc | **12.30** | 1.08 | 0.53 | - | - |
| * Twitch-2 | - | - | - | * 10.00 | - |
| * Twitch-3 | - | - | - | - | * 7.00 |

**Supplementary Table 1.** Dynamic ranges of the various BRET constructs. The dynamic range was calculated by the formula $(R_i-R_0)/R_0$ where $R_i$= BRET ratio at high $Ca^{++}$ (50 µM $Ca^{++}$) and $R_0$=BRET ratio at low $Ca^{++}$ (10 mM EGTA without any added calcium).

*The values for the original Twitch-2 and Twitch-3 FRET sensors were based on published values from purified protein samples[1].

| | |
|---|---|
| Troponin C sequence | GACCAACGCATGCAAGATGCTAGCGAAGAAGAGCTATCAGAGT GCTTTCGCATTTTTGACAAGGATGGAAATGGTTTCATCGACAGG GAGGAATTTGGAGACATCATCCGCCTTACTGGAGAACAGCTCAC AGATGAGGATCCCGATGAGATATTTGGAGATTCAGACACAGACA AAAATGGAAGGATTGACTTTGATGAGTTCCTGAAGATGGTGGAG AATGTCCAGCCTCTGGCCGAGCTGCACTG |
| T2A sequence | GGCAGTGGAGAGGGCAGAGGAAGTCTGCTAACATGCGGTGACG TCGAGGATAATCCTGGCCCC |

**Supplementary Table 2. Nucleotide sequences of TnC and P2A domains.**

| | |
|---|---|
| Venus-*EcoRI*(Kozak)-F | ATCG*GAATTCGCCACC*ATGGTGAGCAAGGGCGAGGAGCTGTTCACC |
| Venus-*EcoRI*-F | ATCG*GAATTC* ATGGTGAGCAAGGGCGAGGAGCTGTTCACC |
| Venus-*SacI*-R | ATCG*GAGCTC*CTTGTACAGCTCGTCCATGCCGAGAGTG |
| Venus(CD10)-*SphI*-R | CTT*GCATGC*GGGCGGCGGTCACGAACTCCAGCAGGACCA |
| T2A-NLuc-*XhoI*-R1 | GACCTCGAGTGGGCCAGGATTCTCCTCGACGTCACCGCATGTTAGCAG ACTTCCTCTGCCCTCTCCAC |
| T2A-NLuc-R2 | AGCAGACTTCCTCTGCCCTCTCCACTGCCGAGCTCCGCCAGAATGCGTT CGCACAGCC |
| Cheriff-*XhoI*-F | TGAC*CTCGAG*GGCGGAGCTCCTGCTCCAGACGCTCACAGCGCCCC |
| Cheriff-*KpnI*-R | TGAC*GGTACC*TTACACGTTGATGTCGATCTGGTCCAGGGGG |
| Melanopsin-*XhoI*-F | TGAC*CTCGAG*ATGAACCCTCCTTCGGGGCCAAGAGTCCCGCCCAGCCC |
| Melanopsin-*KpnI*-R | TGAC*GGTACC*AAGCTTACTACATCCTGGGGTCCTGGCTGGGGATCAGCC |
| NLuc-*KpnI*-R(stop) | CTGT*GGTACC*TTACGCCAGAATGCGTTCGCACAGCCGCC |
| NanoLuc-*HindIII*-R | ATCG*AAGCTT*ACGCCAGAATGCGTTCGCACAGCCGCC |
| Venus(CD10)-*SphI*-R | CTT*GCATGC*GGGCGGCGGTCACGAACTCCAGCAGGACCA |
| TnC-*SphI*-F | CGTGACCGCCGCCC*GCATGC*AAGATGCTAGCGAAGAAGAGC |
| Venus157-*SphI*-R | AGTC*CATGC*G CTGCTTGTCGGCGGTGATATAGACGTTG |
| Venus157-*EcoRI*-F | AGTC*GAATTC* ATGAAGAACGGCATCAAGGCCAACTTCAAGA |
| Venus173-*EcoRI*-F | AGTC*GAATTC* ATGGGCGGCGTGCAGCTCGCCGACCACTAC |
| Venus173-*SphI*-R | AGTC*GCATGC*G GTCCTCGATGTTGTGGCGGATCTTGAAG |
| cpNLuc50-*SacI*-F | AGTC *GAGCTC* GAAAATGGGCTGAAGATCGACATCC |
| cpNLuc50-*HindIII*-R | AGTC *AAGCTT*A ACCGCTCAGGACAATCCTTTGGATC |
| cpNLuc87-*SacI*-F | AGTC *GAGCTC* CATCACTTTAAGGTGATCCTGCACTA |
| cpNLuc87-*HindIII*-R | AGTC *AAGCTT*A ATCATCCACAGGGTACACCACCTTAA |
| cpNLuc136-*SacI*-F | AGTC *GAGCTC* GGCAACAAAATTATCGACGAGCGCCT |
| cpNLuc136-*HindIII*-R | AGTC *AAGCTT*A GTTCCACAGGGTCCCTGTTACAGTGA |
| cpNLuc148-*SacI*-F | AGTC *GAGCTC* GGCTCCCTGCTGTTCCGAGTAACCATC |
| cpNLuc148-*HindIII*-R | AGTC *AAGCTT*A GTCGGGGTTGATCAGGCGCTCGTCGA |

**Supplementary Table 3. Primers for PCR cloning. Restriction enzyme sites are italicized.**

## Supplementary Methods

### Construction of plasmids

Each fusion protein was constructed by polymerase chain reaction (PCR) cloning using pVenus-N1 (Addgene ID#: 61854) and pNL1.1 (Promega©) as template DNA for the Venus and Nanoluc (NLuc) moieties respectively, and the Troponin C (TnC) moieties[1] were created by overlap PCR (primers and sequences in Supplementary Tables 2 and 3). The effect of circular permutations of both NanoLuc (GGGGGSGGGGT linker) and Venus was tested by splitting Venus at amino acid positions 157 and 173[2], while NanoLuc was split at positions 50, 87, 136 and 148. The TnC domain was positioned between Venus (N-terminal) and Nluc (C-terminal). TnC conformational changes due to $Ca^{++}$ binding changed the molecular distance between NanoLuc and Venus, thereby allowing and/or modulating bioluminescence resonance energy transfer (BRET) in response to various $Ca^{++}$ concentrations [$Ca^{++}$]. The CalfluxVTN fusion protein was cloned into plasmids pRSETB (Invitrogen) and pCAG (from VSFP Butterfly 1.2[3]) {Addgene #: 47978} for bacterial and mammalian expression, respectively. For adeno-associated viral (AAV) transduction, CalfluxVTN and CalfluxVTN-2A-CheRiff were cloned into AAV pCAG-FLEX-tdTomato-WPRE (Addgene #: 51503) and the plasmid was shipped to ViGene Biosciences (Rockville, MD, USA) where they created a mix of serotype 1 + 2 AAV particles. The AAV tdTomato plasmid was first modified to introduce the restriction enzyme sites for EcoR1 and HindIII and then the desired $Ca^{++}$-sensor was ligated into the open reading frame.

### Protein purification and *in vitro* experiments

Fifteen different combinations of NanoLuc and Venus (including circularly permuted versions) of the BRET $Ca^{++}$ sensor were constructed and the purified fusion proteins tested *in vitro* (Supplementary Fig. 1-2). The constructs were inserted into pRSETB, and the BRET ratio (signal at 525 nm divided by signal at 450 nm, see below) of the fusion proteins (isolated via their His6 tags, see below) were measured in high [$Ca^{++}$] (50 μM $CaCl_2$) and low [$Ca^{++}$](10 μM EGTA) buffer (100 mM KCl, 30 mM MOPS, pH 7.2). The fusion protein with the largest dynamic range was Venus-TnC-Nluc (Supplementary Fig. 1a), and was renamed CalfluxVTN for "CALcium FLUX composed of Venus (V), Troponin (T), and NanoLuc (N)." Using the EcoRI (upstream) and HindIII (downstream) restriction enzyme sites, CalfluxVTN was inserted into pRSETB, then was transfected into BL21 *E. coli* for expression and purification via an N-terminally linked, six-histidine-residue tag (His6). The T7 tag and the enterokinase sites were excised from pRSETB so that the CalfluxVTN sequence is immediately adjacent to the His6 sequence. The His6-tagged fusion protein was purified via TALON® Metal Affinity $Co^{++}$ Resin and the BRET signal, in response to varying [$Ca^{++}$], was measured in $Ca^{++}$ buffers from Molecular Probes® (Life Technologies™) using a QuantaMaster™ (Photon Technology International Inc.) fluorescence spectrophotometer with the excitation off. BRET ratiometric values were calculated by dividing the light emitted at 525 nm by that emitted at 450 nm after a full scan of the spectrum from 400 to 600 nm. Similarly, the BRET signal of purified CalfluxVTN was also measured microscopically using a hemacytometer to ensure a uniform volume of the protein solution. The BRET ratio was measured using emission filter cube sets of 480/40 nm bandpass (NanoLuc luminescence peak) and 520 LP (Venus fluorescence peak) in the microscopic apparatus described below under "Microscopic Imaging." For all experiments, the NanoLuc substrate, furimazine, was added to a final concentration of 10 μM.

### Cellular Expression of $Ca^{++}$-Sensors and Recording

The plasmid VSFP Butterfly 1.2 was used as the backbone for the expression of CalfluxVTN in mammalian cells. CalfluxVTN was inserted into the backbone via EcoRI and KpnI sites in order to drive expression by the CAG promoter[4]. (A Kozak sequence[5] was included downstream of the CAG promoter and just upstream of the CalfluxVTN gene). Cell lines were HEK293 (ATCC#CCL-2™) and HeLa (ATCC#CRL-1578™). CalfluxVTN was transfected into HEK293 or HeLa cells via Lipofectamine 2000 (ThermoFisher Scientific Inc.) or GeneCellin (BioCellChallenge) *in vitro* transfection reagents by the manufacturers' instructions. HEK293 and HeLa cells were imaged in the following recording medium (in mM): 1.26 $CaCl_2$, 0.49 $MgCl_2$, 0.41 $MgSO_4$, 5 KCl, 0.44 $KH_2PO_4$, 4.16 $NaCO_3$, 150 NaCl, 0.34 $NaHPO_4$, 10 HEPES and 0.6 % wt/vol D-glucose. To assay changes in cytosolic $Ca^{++}$ via CalfluxVTN's BRET signal during live-cell imaging, 1 μM Thapsigargin (TG) or 10 μM ionomycin were added to HEK293 cells. To initiate cytosolic $Ca^{++}$ oscillations during live-cell imaging, 10 μM Histamine was added to HeLa cells extracellularly. To compare $Ca^{++}$-induced responses of CalfluxVTN vs. GCaMP6s, the $Ca^{++}$-sensors (CalfluxVTN and GCaMP6s) were expressed separately in HEK293 cells and the cells were incubated in the recording medium (above) with varying $CaCl_2$ concentrations (1 mM, 5 mM, 10 mM $CaCl_2$). The cells were then imaged on the microscope and their responses to 10 μM ionomycin were recorded using either luminescence microscopy (CalfluxVTN) or epi-fluorescence microscopy (GCaMP6s).

To test CalfluxVTN's responses in neurons, hippocampal neurons were isolated from day 18 rat embryos (Sprague Dawley rats, Charles River Laboratories Inc.), cultured at low density, and transfected via DNA-$Ca_3(PO_4)_2$ precipitation as described previously[8-10] with 4-8 µg of plasmids encoding CalfluxVTN under the control of the CAG promoter {pCAG[4]}. At DIV 7-8 the neurons were transfected with the relevant DNA constructs. At DIV 10-14, the neurons that were positive for Venus fluorescence were imaged for their responses to the varying experimental perturbations. Neurons from these rats were also transfected via AAV particles (serotype AAV 1 + 2 mix). Pre-made AAV particles (ViGene Biosciences, MD, USA) were diluted in phosphate buffered saline (PBS; Gibco™) and added to neurons after 1 week in culture. For testing the response of the neurons to varying concentrations of extracellular $K^+$, the medium surrounding the neurons was exchanged with test buffer (20 mM HEPES pH 7.2, 10 mM D-glucose, 2 mM $CaCl_2$, 1 mM $MgCl_2$, 1 mM $Na_2HPO_4$, 134 mM NaCl, and 5 mM KCl. This standard test buffer was then exchanged manually within the imaging chamber with test buffers of the same composition except the [KCl] and [NaCl] were reciprocally varied to produce the desired isotonic solutions with different potassium concentrations.

**Compatibility with Optogenetic Rhodopsins**

Two bi-cistronic expression plasmids were created: CalfluxVTN plus mouse melanopsin (*opn4*) under the control of pCAG[4], and GCaMP6s plus Opn4 under the control of pCAG[4], each separated by the T2A sequence[9]. Similarly, another bi-cistronic expression vector was constructed with the channelrhodopsin variant CheRiff[10] {Addgene #:51695) replacing *opn4*[11]. These were transfected (see Cellular Expression and Characterization) into cells (HEK293 cells or primary hippocampal neurons) and stimulated with ~470 nm (470/30 nm) light for the length of time depicted in each figure. A custom journal was defined in Metamorph Premier® so that illumination and image acquisition could be carefully controlled.

**Hippocampal viral injection and brain slice preparation**

On the day of surgery, male C57BL/6J mice were anesthetized with isoflurane (3% initial, 1.5% for maintenance) and placed in a stereotaxic apparatus (my NeuroLab, Leica AngleTwo stereotaxic system; Leica Biosystems, BuffaloGrove, IL). Angle Two software was used for setting injection targets in the dorsal hippocampus (coordinates were 2.18 mm posterior to bregma, 2.78 mm lateral to the midline, and 1.73 mm below the skull surface). Other details about the surgical procedure have been described previously[12]. A 33-gauge needle of a 10 µl syringe (Hamilton Company, Reno, NV) was heat sterilized immediately before back filling with AAV-CalfluxVTN virus. 500 nL of the virus was injected bilaterally into the dorsal hippocampus at 50 nL/min using a UltraMicroPump II and Micro4-controller (World Precision Instruments, Sarasota, FL). Five minutes later, the syringe was withdrawn and the scalp wound was sutured. Postsurgical care included immediate subcutaneous saline (1.0 ml per 20 g of body weight) and analgesic (ketoprofen, 5 mg/kg, subcutaneously) followed by additional ketoprofen injections every 24 hours for 3 days. Animals were monitored for health concerns including loss of body weight >20%, signs of uncontrolled pain, stress or dehydration. No animals displayed these signs and therefore none were removed from further studies. All animal studies were performed under guidelines approved by Vanderbilt University's Institutional Animal Care and Use Committee (IACUC).

Brain slice preparation was performed as previously described[13,14]. Mice were removed from the colony and allowed to acclimate for 1 h in a Med Associates sound-attenuating chamber. After the acclimation, mice were anesthetized with isoflurane and then decapitated. Brains were removed quickly and transferred to a 1°C–4°C oxygenated, low sodium sucrose dissecting solution (concentrations in mM: 183 sucrose, 20 NaCl, 0.5 KCl, 2 $CaCl_2$, 1 $MgCl_2$, 1.4 $NaH_2PO_4$, 10 glucose, 26 $NaHCO_3$). A Leica vibratome was used to prepare coronal brain slices (300 µm) containing the hippocampus. Slices were then transferred to a holding chamber for recordings. The holding chamber contained heated, oxygenated ACSF (119 mM NaCl, 26.2 mM $NaHCO_3$, 2.5 mM KCl, 1 mM $NaH_2PO_4$, 1.3 mM $MgCl_2$, 10 mM D-glucose, 2.5 mM $CaCl_2$; sterile filtered).

**Flow-through microscopic imaging experiments of acute brain slices**

The acute brain slices were prepared as described above and then kept in ACSF (119 mM NaCl, 26.2 mM $NaHCO_3$, 2.5 mM KCl, 1 mM $NaH_2PO_4$, 1.3 mM $MgCl_2$, 10 mM D-glucose, 2.5 mM $CaCl_2$; sterile filtered) at 37 ºC, while bubbled continuously with 95% $O_2$ - 5% $CO_2$. In an open flow chamber (Warner Instruments Inc.: RC-26G), a gravity flow system was set up where the inward flow (2 ml/min) was controlled by gravity but the outward flow was removed by a peristaltic pump (Pharmacia©: LKB-Pump P-1). The ACSF was bubbled with 95% $O_2$ - 5% $CO_2$ before it flowed into the chamber. Because imaging occurred on an inverted microscope, a small nylon mesh was placed between the surface of the acute brain tissue and the glass coverslip that formed the bottom of the chamber,

to allow for good oxygenation of both the top and bottom of the slice. A metal Harp (Warner Instruments Inc.: SHD-26GH/10) kept the slice in place during imaging.

**Microscopic Imaging and Data Analysis**
Primary neurons, acute tissue slices, and immortalized cells were imaged on an inverted Olympus IX-71 epi-fluorescence microscope inside a temperature-controlled, light-tight box. A liquid-cooled EB-CCD (Hamamatsu Photonics K.K., C7190-13W) was used to image the cells at a frame rate of 1-4 Hz. To capture and ratio the BRET images, filters for NanoLuc (EM 480/40 nm bandpass) and Venus (EM 520 longpass) were rotated with a motorized filter turret wheel within the microscope to alternately image the blue vs. yellow/green wavelengths. The speed of rotation of these filters was ~100 ms. For faster imaging (e.g., Fig. 9d), an EM-CCD camera (Hamamatsu ImagEM X2) was used with comparable results. This EM-CCD camera was coupled to a light splitter (Hamamatsu W-View Gemini) that used a dichroic filter (495 nm LP) to separate the blue and yellow light and projected them onto distinct regions of the camera's CCD chip. This allowed for simultaneous collection of blue and yellow wavelengths for optimal temporal resolution. During luminescence microscopy, all images were collected in complete darkness. To stimulate the Opn4 or the CheRiff optogenetic probes, ~470 nm (470/30 nm) light was used for the duration stated in the relevant figure legend. Neurons that were cultured on glass coverslips (ThermoFisher™) were inserted into a Chamlide magnetic chamber (Live Cell Instrument, Korea) for imaging.

The images were analyzed with ImageJ software (NIH) using background-subtracted images collected from the blue and yellow channels. After background subtraction, a simple division of the yellow wavelength intensities by the blue wavelengths produced the BRET Ratio values as depicted in all figures. The average light intensity of blue vs. yellow from regions of interest within the cells was compared pixel by pixel to obtain ratiometric BRET estimates of cytosolic $Ca^{++}$ over the image. To create the ratiometric photos and movies (e.g., Fig. 5b), both blue and yellow channels were background subtracted using the ImageJ function. Then the blue wavelength images were used as a template to create an image that separated the cells completely from the background (cell region was given a value of 255, and background was given 0). The blue and yellow images were then multiplied by this template image where the background was held at 0 and areas within cells were given the full bit value. The resulting images were converted from 16-bit (from camera) to a 32-bit to mitigate losing data after the multiplication step. To finally achieve the ratiometric image, the yellow emission needed to be divided by the blue emission, but to prevent dividing by 0, a gray value of 1 (negligible, since it is out of $4.3 \times 10^9$) was added to all the pixels in the blue images, so that dividing the yellow values by the blue values would not result in division by 0 at any pixel. When the final yellow images were divided by the final blue images, the ratio changes that are indicative of $[Ca^{++}]$ were observed (as indicated by the calibration curve in Fig. 1b-c or in Supplementary Fig. 8). The ImageJ lookup table (LUT) named "fire" was applied to the images to better highlight the areas were $[Ca^{++}]$ was changing.

Statistical significance was tested using the Data Analysis Tool Pack from Microsoft Excel (Microsoft©). The Hill coefficient and $K_d$ values (Fig. 1c) were determined using OriginLab 6 software (OriginLab©). The emission light spectrophotometric data measured with the QuantaMaster (Figs. 1 & 2) were collected across the 400-600 nm range in 1-nm intervals. The microscopic data were collected as the average pixel intensity over a user-defined region of interest (ROI). Where t-tests were done, the data fit a normal distribution (Fig. 2d, 3d, 4e-f) and for non-parametric data sets (Fig. 5d, 7d, 9c) ANOVAs were used to determine the significant difference between experimental groups. Except for the negative control neurons in Fig. 7d, statistical analyses were performed in cellular experiments that contained a minimum of 10 cells (in a single experiment) or a minimum of 3 independent experiments, with multiple cells from each group. Unhealthy cells were identified as those cells whose BRET ratio exceeded 3 before any experimental manipulation was done, and were excluded from further analyses.

Note that the BRET ratio scale as a function of $[Ca^{++}]$ ranges over 1.0-12.1 for the QuantaMaster™ measurements (Fig. 1b-c), but the same calibration curve generated with purified CalfluxVTN and $Ca^{++}$ buffers from Molecular Probes® on the microscopic setup ranges over 1.0-10.5 (Supplementary Fig. 8). This difference is due to the BRET ratio from QuantaMaster™ measurements being calculated from peak values of the blue emission (~450 nm) vs. the peak values of the yellow emission (~525 nm, Fig. 1b-c), whereas the BRET ratio calculated from the microscopic setup includes all blue emission in the range of 440-520 nm vs. all emission of wavelengths longer than 520 nm (Supplementary Fig. 8). Additionally, a higher protein concentration was required for microscopy, due to reduced sensitivity of the EB-CCD camera compared to the QuantaMaster™. These differences cause a substantial difference in the BRET ratio range for any particular $[Ca^{++}]$. Because the imaging data illustrated in Figs. 3-9 was generated from microscopic measurements, the BRET ratio calibration curve depicted in Supplementary Fig. 8

should be used to evaluate [$Ca^{++}$] levels in those experiments. However, much of the literature reporting BRET ratio uses an *in vitro* assay as in Fig. 1, and therefore the calibration curve in Fig. 1c (= "QuantaMaster" curve in Supplementary Fig. 8) is included for making comparisons to other literature (note that the *in vitro* assay is also relevant to Fig. 2, Supplementary Figs. 1 and 2, and Supplementary Table 1).

## Supplementary References


1. Thestrup, T., et al. Optimized ratiometric calcium sensors for functional in vivo imaging of neurons and T lymphocytes. *Nat. Methods* **11**, 175-182 (2014).

2. Nagai, T., Yamada, S., Tominaga, T., Ichikawa, M., & Miyawaki, A. Expanded dynamic range of fluorescent indicators for $Ca^{2+}$ by circularly permuted yellow fluorescent proteins. *Proc. Natl. Acad. Sci. USA* **101**, 10554–9 (2004).

3. Akemann, W., et al. Imaging neural circuit dynamics with a voltage-sensitive fluorescent protein. *J. Neurophysiol*. **108**, 2323-2337 (2012).

4. Niwa, H., Yamamura, K., & Miyazaki, J. Efficient selection for high-expression transfectants with a novel eukaryotic vector. *Gene* **108**, 193–199 (1991).

5. Kozak, M. Point mutations close to the AUG initiator codon affect the efficiency of translation of rat preproinsulin in vivo. *Nature* **308**, 241–246 (1984).

6. Kaech, S., & Banker, G. Culturing hippocampal neurons. *Nat. Protoc.* **1**, 2406–2415 (2006).

7. Jiang, M., & Chen, G. High $Ca^{2+}$-phosphate transfection efficiency in low-density neuronal cultures. *Nat. Protoc*. **1**, 695-700 (2006).

8. Lin, W.H., Nebhan, C.A., Anderson, B.R., & Webb, D.J. Vasodilator-stimulated phosphoprotein (VASP) induces actin assembly in dendritic spines to promote their development and potentiate synaptic strength. *J. Biol. Chem*. **285**, 36010-36020 (2010).

9. Kim, J.H., et al. High cleavage efficiency of a 2A peptide derived from porcine teschovirus-1 in human cell lines, zebrafish and mice. PLoS One **6**, e18556 (2011).

10. Hochbaum, D.R., et al. All-optical electrophysiology in mammalian neurons using engineered microbial rhodopsins. *Nat. Methods* **11**, 825-833 (2014).

11. Qiu, X., et al. Induction of photosensitivity by heterologous expression of melanopsin. *Nature* **433**, 745-749 (2005).

12. Kash, T.L. & Winder, D.G. Neuropeptide Y and corticotropin-releasing factor bi-directionally modulate inhibitory synaptic transmission in the bed nucleus of the stria terminalis. *Neuropharmacology* **51**, 1013-22 (2006).

13. Flavin, S.A., Matthews, R.T., Wang, Q., Muly, E.C. & Winder, D.G. alpha(2A)-adrenergic receptors filter parabrachial inputs to the bed nucleus of the stria terminalis. *J Neurosci* **34**, 9319-31 (2014).

14. Silberman, Y., Fetterly, T.L., Awad, E.K., Milano, E.J., Usdin, T.B. & Winder, D.G. Ethanol produces corticotropin-releasing factor receptor-dependent enhancement of spontaneous glutamatergic transmission in the mouse central amygdala. *Alcohol Clin Exp Res* **39**, 2154-62 (2015).
